# Functional coatings for textiles: advancements in flame resistance, antimicrobial defense, and self-cleaning performance

**DOI:** 10.1039/d5ra01429h

**Published:** 2025-04-08

**Authors:** Joyjit Ghosh, Nishat Sarmin Rupanty, Tasneem Noor, Tanvir Rahman Asif, Tarikul Islam, Vladimir Reukov

**Affiliations:** a Department of Textiles, Merchandising, and Interiors, University of Georgia Athens Georgia 30602 USA tarikul@uga.edu; b Department of Textile Engineering, Ahsanullah University of Science and Technology Dhaka 1208 Bangladesh; c Department of Textile Engineering, Jashore University of Science and Technology Jashore 7408 Bangladesh

## Abstract

The continuous evolution of textile technologies has led to innovative functional coatings that enhance protective textiles by integrating flame retardancy, antimicrobial efficacy, and self-cleaning properties. These multifunctional coatings address the growing demand for high-performance materials in healthcare, military, and industrial applications. This study reviews advancements in coating techniques, including dip-coating, spray-coating, sol–gel processes, and layer-by-layer assembly, highlighting their effectiveness in imparting durability, thermal stability, and biological activity to textile substrates. The incorporation of bioactive materials such as chitosan, silver nanoparticles, and plant-derived antimicrobials has demonstrated enhanced pathogen resistance and prolonged fabric functionality. Furthermore, recent developments in phosphorus-based flame retardants and photocatalytic self-cleaning agents, including titanium dioxide and silica nanoparticles, have contributed to the sustainability of functional textiles by reducing environmental impact. Challenges remain in achieving compatibility among diverse functional components while maintaining mechanical integrity and user comfort. Scalability and cost-efficiency also present barriers to commercialization, necessitating cross-disciplinary collaboration among material scientists, engineers, and regulatory experts. Future research should focus on biodegradable alternatives, smart-responsive coatings, and advanced nanomaterial integration to enhance the longevity and eco-friendliness of protective textiles. As industry standards shift towards sustainability, functional coatings are poised to redefine textile applications, offering tailored solutions that balance safety, performance, and environmental responsibility. This review underscores the transformative potential of multifunctional textile coatings and their role in advancing next-generation protective fabrics.

## Abstract 2

A new generation of functional coatings offers flame retardancy, antimicrobial efficacy, and self-cleaning properties, making them valuable additions to healthcare, the military, and industrial applications.

## Graphical abstract

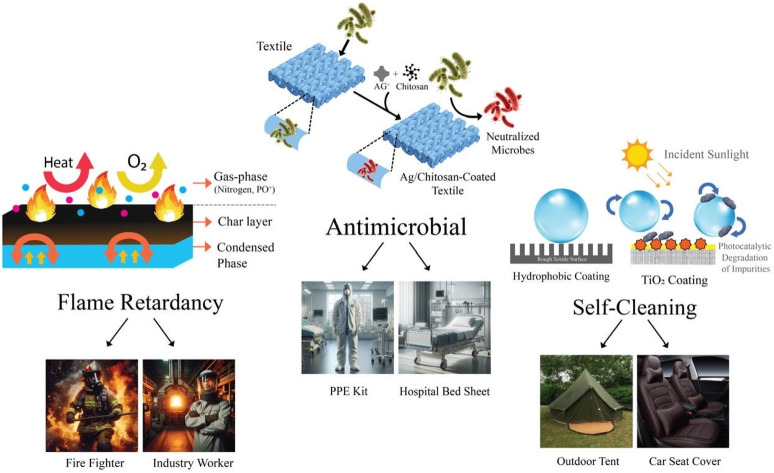

## 1. Introduction

Innovative functional coatings that improve flame retardancy, antibacterial qualities, and self-cleaning capabilities are revolutionizing protective textiles. By enhancing performance, safety, and durability, these developments satisfy the rising need for high-efficiency materials. This review focuses on the latest developments and new directions in functional coatings.

Natural fibers such as cotton, silk, and wool, as well as synthetic fibers like regenerated cellulose, nylon, and polyester, each have their own set of qualities. These materials are chemically and physically modified to improve functions such as antibacterial, water-repellent, and flame-retardant qualities for advanced textile applications.^1^ Synthetic and natural fibers are utilized in coatings and composites, influencing the materials' performance, strength, and flexibility. Increasingly, bio-based elements are being included into textile reinforcements and coatings to improve functionality and sustainability.^2^ Wool is a natural protein-based textile substrate that is fire retardant, stain resistant, and warm, making it suitable for clothing and flooring. Various treatments, including enzymatic, polymeric coatings, and plasma treatments, are used to improve shrink resistance and durability while being sustainable.^3^ Cotton, polyethylene terephthalate (PET), and nonwoven textile substrates are frequently utilized in antimicrobial applications; therefore, they need to be modified to improve their durability, antibacterial qualities, and filtering effectiveness. For technological and medical applications, a variety of surface functionalization processes are used to enhance their performance and protective qualities.^4^ Polyethylene terephthalate (PET) is a popular synthetic textile substrate noted for its durability but lacks perspiration absorption and antistatic qualities. Modifications such as saponification and biopolymer treatments improve its hydrophilicity, UV protection, and comfort while maintaining mechanical integrity.^5^

Due to the increasing exposure to infectious diseases by healthcare workers during the COVID-19 pandemic, there has been a major increase in demand for protective textiles in the healthcare industry. A vital line of defense against infections is personal protective equipment (PPE), which includes gloves, masks, and gowns. The circumstance calls for advancements in material science and production techniques while highlighting the critical role protective apparel plays in preserving the health of healthcare personnel.^6^ The need for high-performance protective fabrics that successfully stop liquid and microbiological transmission is highlighted by the rise in newly developing infectious illnesses.^7^ To defend soldiers from ballistic, biological, and chemical threats, the military employs cutting-edge technologies. These materials protect industrial workers from dangerous conditions like flames and toxins. There will likely be a greater need for protective fabrics as people become more conscious of safety and protection, which will encourage innovation in this crucial area.^8^ Protective fabrics are crucial for industries, the military, and healthcare because they keep people safe and comfortable in dangerous settings. They ensure sterile conditions in healthcare by guarding against chemicals and biological pollutants. To prevent thermo-physiological strain, they assist employees in avoiding chemical and physical risks while preserving their comfort in industrial environments. Because of stricter safety regulations and increased awareness of the health hazards associated with different jobs, there is a growing need for sophisticated protective fabrics.^9^ In the healthcare, military, and industrial sectors—where usefulness and safety are critical—protective fabrics are indispensable. They offer medical staff barriers against infections in the healthcare industry. These materials are used by the military to make clothing and equipment that provide chemical and ballistic protection. Wearing protective gear in industrial environments ensures that safety rules are followed while protecting workers from chemicals and mechanical risks. The development of protective textiles has been fueled by growing workplace safety awareness, leading to developments that blend utility, comfort, and durability to satisfy the various demands of different industries.^10^

Specialized finishes called functional coatings are applied to textiles to provide qualities that improve their functionality and performance. These coatings improve textile items' comfort, safety, and durability, among other things.^11^ Coatings change the fabric's surface properties without affecting its inherent attributes. Their goal is to turn traditional fabrics into materials with multiple uses that can react to environmental cues or offer defensive advantages. For example, functional coatings can make textiles fire-resistant, water-repellent, or antibacterial, expanding their utility in everyday, medicinal, and technological applications. Click or tap here to enter text. When altering important textile characteristics to satisfy performance standards, functional coatings are essential. For example, hydrophobic treatments that stop water absorption give fabrics water repellency, which makes them appropriate for outdoor and weather-resistant uses. Another crucial change is fire resistance, which involves applying coatings to textiles to lessen their flammability and boost safety in areas where fire threats are a problem. Particularly in medical and athletic clothing, antimicrobial coatings prevent the growth of bacteria and fungi on textiles, enhancing longevity and hygiene. UV protection treatments protect textiles from damaging UV radiation, protecting the material's integrity and the wearer. Furthermore, sophisticated coatings that make it simple for dirt and stains to be removed can be used to add self-cleaning properties, which lowers maintenance requirements and improves the usefulness of the fabrics.^11^ By altering these characteristics, functional coatings increase the usefulness and durability of textiles while enabling them to satisfy the requirements of contemporary applications in various industries, such as clothing, outdoor equipment, and medical textiles.^12^ Furthermore, antimicrobial coatings promote hygiene in sporting and medical fabrics by preventing the growth of bacteria and fungi. UV protection coatings prolong the life of textiles exposed to sunshine by shielding the fabric from damaging UV rays. To preserve the beauty of fabric and lower maintenance requirements, self-cleaning properties are provided through coatings that help dirt and stains break down when exposed to light or moisture. Textiles can function in various settings through these changes, extending their useful life.^13^

A methodical criterion for including research on functional coatings for protective textiles was developed during the review process. Scientific rigor, publication recentness (within the previous five years),^14^ and relevance to the main functionalities of interest—flame retardancy, antimicrobial qualities, and self-cleaning mechanisms—were the criteria used to choose the examined studies. To reduce any bias and improve the dependability of the insights offered in this review, this methodology was used. The objectives of this review are multifaceted and aim to provide a comprehensive understanding of the advancements in functional coatings for protective textiles. First, it seeks to assess new developments within the field, highlighting recent innovations that enhance textile performance. Additionally, the review will determine the main obstacles that hinder further progress, such as technical challenges or regulatory barriers. Another key objective is to evaluate real-world applications of these coatings across various sectors, showcasing their practical utility and effectiveness in everyday scenarios. The review will also examine coating methods in applying these functionalities to textiles, providing insights into their efficacy and scalability. Furthermore, an essential aspect of this investigation involves exploring how multifunctional coatings can be effectively integrated to achieve synergistic benefits without compromising performance. As sustainability becomes increasingly important, considering safety and environmental considerations is paramount; thus, this review will delve into eco-friendly practices associated with functional coatings. Lastly, the review aims to offer suggestions for additional research avenues that could further contribute to advancing knowledge and innovation in this dynamic area. Through these objectives, the review aspires to illuminate current trends and foster future growth within the protective textile industry.

In this study, functional coatings designed specifically for protective fabrics are thoroughly examined. Included in the scope is an overview of the various methods for applying functional coatings, including dip-coating, spray-coating, plasma treatment, sol–gel processes, and layer-by-layer assembly. Functional coatings offers a comprehensive analysis of flame-retardant, antimicrobial, and self-cleaning coatings, covering their fundamental components, performance evaluation, and recent advancements. Investigate the potential and challenges of combining several uses into a single coating system, analyze the effects of these coatings on people's health and the environment, follow rules, and search for sustainable alternatives.

## 2. Coating techniques for functional textiles

### 2.1 Overview of coating techniques

Functional coatings are essential for improving textile performance by providing attributes like flame retardancy, antimicrobial properties, and self-cleaning features. A range of coating techniques has been established to attain these functionalities, each possessing distinct characteristics, benefits, and constraints.

The primer layer functions as a binder, ensuring a strong bond between the textile substrate and the polymer coating. It promotes consistent pigment distribution, increases durability, and preserves oil and water repellent qualities. An excellent primer maintains coating integrity in functional textiles using the squeegee process, ensuring long-term stability.^15^ Furthermore, the primer improves adhesion efficiency, allowing the coating to adhere firmly to the textile substrate while reducing cracking and peeling. It also serves as a barrier layer, increasing the bonding strength between the textile and the polymer coating, resulting in uniform coverage and long-lasting durability.^16^ The primer layer also acts as a binding agent between the textile or polymer substrate for the flame-retardant coating, ensuring good adhesion and durability. Primers improve coating uniformity, stability, and fire-resistant performance in surface finishing procedures such as layer-by-layer assembly, dip coating, and spray coating while preserving the material's fundamental qualities.^17^ Additionally, primers improve adhesion strength and coating penetration for better performance by optimizing the substrate-coating interaction. Additionally, they promote layer cohesiveness, which lowers the possibility of peeling or cracking and guarantees the flame-retardant coating's long-term stability and efficacy.^18^

**Fig. 1:**
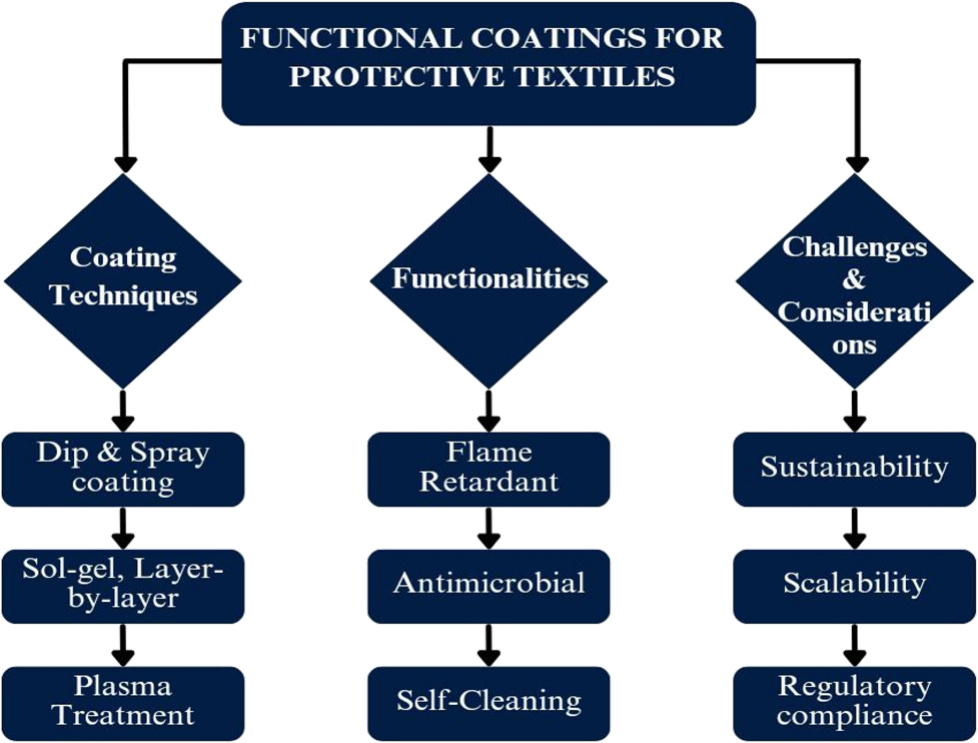
Functional coatings for protective textiles.

Fig. 1 depicts various coating techniques that can be classified according to their application methods and coating characteristics. Common techniques encompass dip-coating, spray-coating, plasma treatment, sol–gel processes, and layer-by-layer assembly.

To ensure uniform coating deposition and enhanced performance in functional applications, surface modification is required to change the textiles' surface energy, roughness, and chemical reactivity. Textile surfaces can be made more useful without changing their basic characteristics by using surface treatment and modification processes as sol–gel processing, electrospinning, and plasma treatment. Water repellency, flame retardancy, and antibacterial performance are all improved by these techniques, guaranteeing sophisticated multipurpose textiles with increased sustainability and longevity.^19^ Surface modification and treatment methods based on plasma improve textile qualities by changing the chemistry of the surface without changing the properties of the bulk material. Advanced textile applications like antimicrobial, water-repellent, and dyeable materials can benefit from these treatments because they increase adhesion, wettability, and functional performance.^20^ Graphene oxide (GO) and reduced graphene oxide (RGO) surface modification improves the electrical conductivity, water resistance, and UV protection of silk textiles. By creating a thin, continuous film through layer-by-layer deposition, surface qualities are enhanced, and durability is preserved even after several washing cycles.^21^

Chemical techniques like grafting, enzymatic modification, and nano-treatments and sophisticated physical procedures like plasma, UV, and ozone treatment are the most current advancements in surface therapy and modification. In addition to providing functional coatings and increased fiber strength for high-performance textile applications, these innovative techniques improve the wettability, shrink resistance, and bacterial resistance of fibers.^22^

### 2.2 Dip-coating

**Fig. 2:**
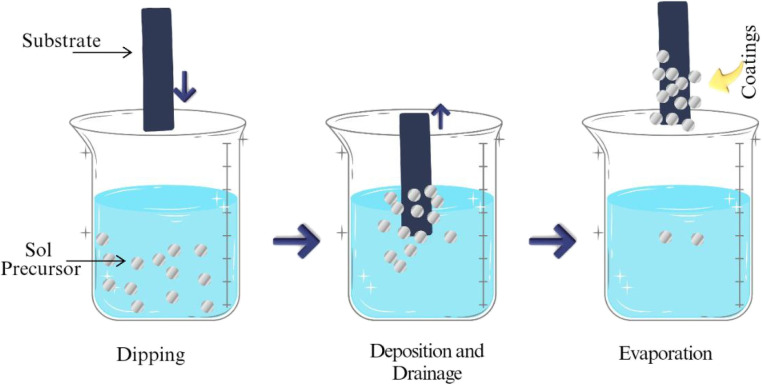
Dip-coating mechanism.

The process of dip-coating entails submerging the textile substrate in a solution that holds the intended coating material. Upon the withdrawal of the textile, the liquid coating bonds to the surface, resulting in a consistent layer.^23,24^Fig. 2 depicts the dip-coating process, showcasing the immersion of a substrate in a coating solution followed by its withdrawal. The quality of the coating is influenced by several parameters, as extended immersion can result in thicker coatings.^25^ Higher temperatures may enhance the interaction between the coating material and textile fibers. Increased concentrations can produce thicker coating layers; however, they might result in inconsistent application. It presents advantages and disadvantages. Effortless configuration, economical, and ideal for extensive manufacturing. Limited to thin coatings, which presents challenges in achieving uniform thickness for complex geometries.^26^

### 2.3 Spray-coating

**Fig. 3:**
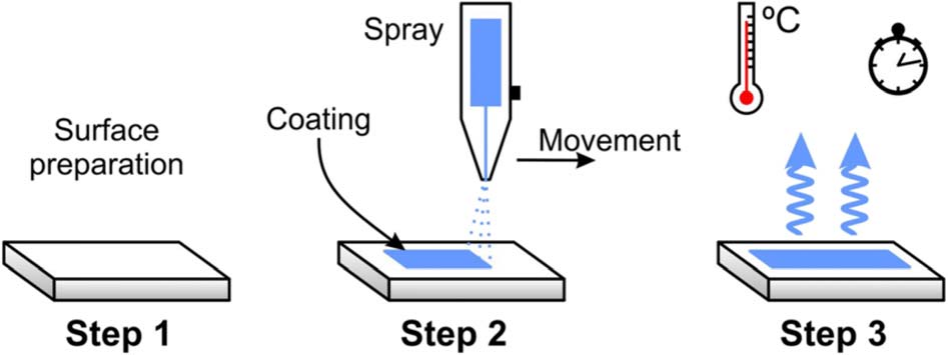
Spray-coating setup. Reproduced with permission from ref. 28 Copyright 2020, Springer-Verlag GmbH Germany, part of Springer Nature.

Spray-coating involves atomizing the coating material and applying it to the textile surface using a spray gun. This method is compatible with various materials and allows for adjustable application rates.^27^Fig. 3 shows a spray used to apply a coating to a textile surface. There are many application considerations, including adjusting the distance from the substrate or the spray pressure can control coating thickness; achieving a uniform coating can be challenging, especially on textured fabrics, and uneven surfaces may lead to varied coating properties.^29^

### 2.4 Plasma treatment

**Fig. 4:**
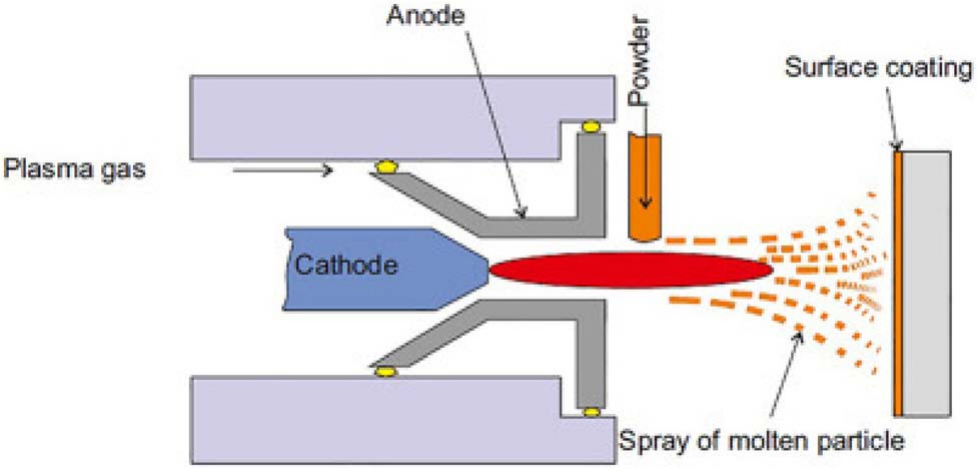
Plasma treatment mechanism. Reproduced with permission from ref. 31 Copyright 2021, Elsevier Ltd.

Plasma treatment modifies the surface chemistry of textiles, enhancing adhesion for subsequent coating applications. This technique involves ionizing gas to create plasma, which interacts with the textile surface.^30^Fig. 4 represents a plasma chamber showing the transition of gas to plasma and its interaction with the textile surface. Usually, plasma has been used to enhance the effectiveness of antimicrobial agents, improve the bonding and performance of flame-retardant coatings, and so on.^32^

### 2.5 Sol–gel process

**Fig. 5:**
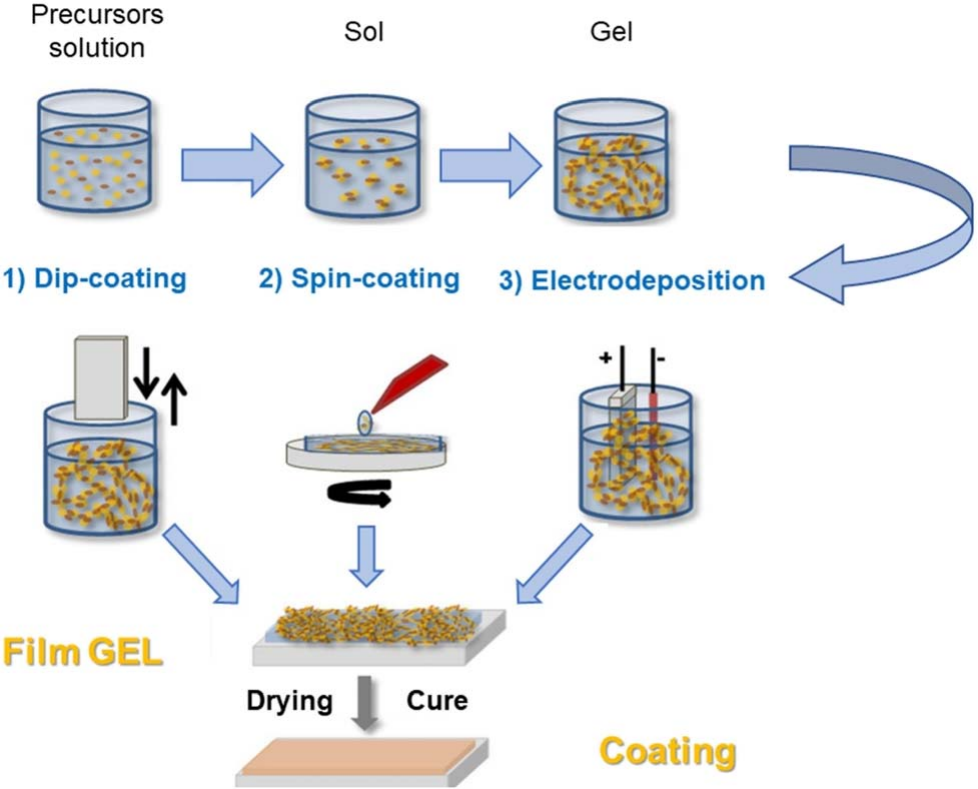
Sol–Gel process overview. Reproduced with permission from ref. 35 Copyright 2022, Elsevier Ltd.

The sol–gel process involves the transition of a solution (sol) into a solid (gel) network. This method can create durable, multifunctional coatings on textiles through controlled hydrolysis and condensation reactions^33,34^Fig. 5 illustrates the sol–gel transition from solution to gel and then to a solid coating on textiles. It produces highly durable coatings with multi-functionality (*e.g.*, hydrophobicity, flame retardancy). However, the process requires many stages to complete individually and takes a long time as well, so the cost and complexity of achieving uniform coatings on large-scale textiles make this process difficult to consider viable.^36,37^

### 2.6 Layer-by-layer assembly

**Fig. 6:**
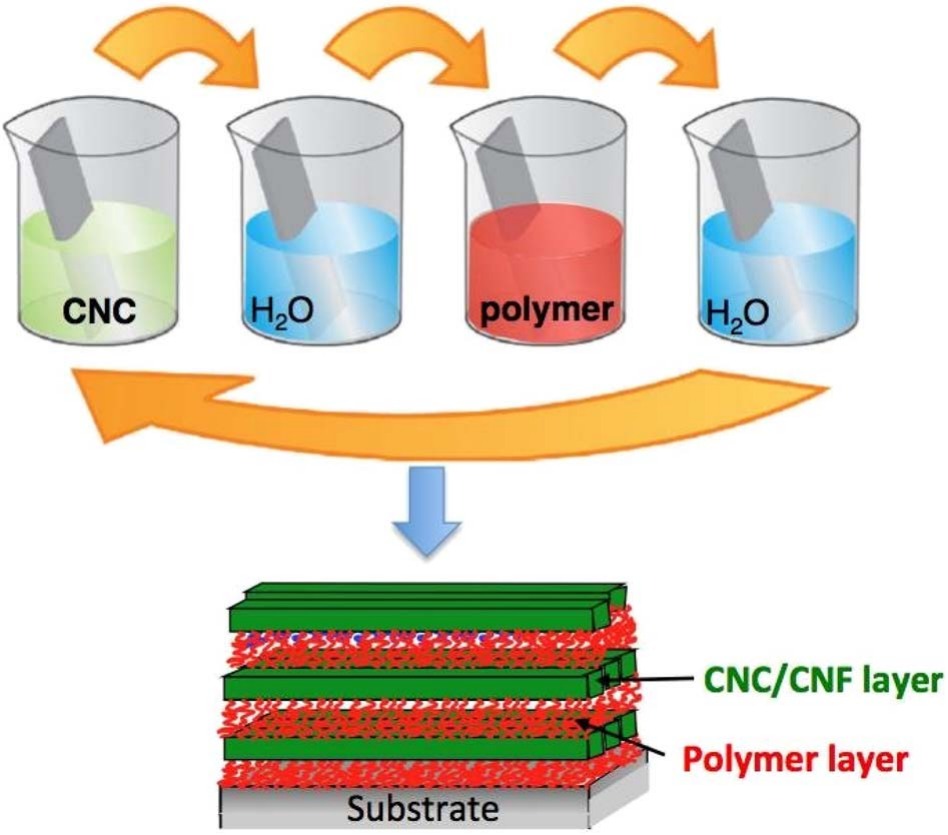
Layer-by-Layer assembly. Reproduced with permission from ref. 39 Copyright 2018, De Gruyter.

The layer-by-layer assembly technique involves sequentially depositing alternating layers of different materials onto the textile surface, allowing for customizable properties and thicknesses.^38^Fig. 6 shows the sequential application of layers onto a textile substrate, highlighting the control over thickness and functionality. Allows for precise control over the functionality and thickness of coatings; potential for creating highly customized textiles. While complexity in scaling production and ensuring uniformity over large surfaces.^38,40^

## 3. Flame retardant coatings

### 3.1 Mechanisms of flame retardancy

**Fig. 7:**
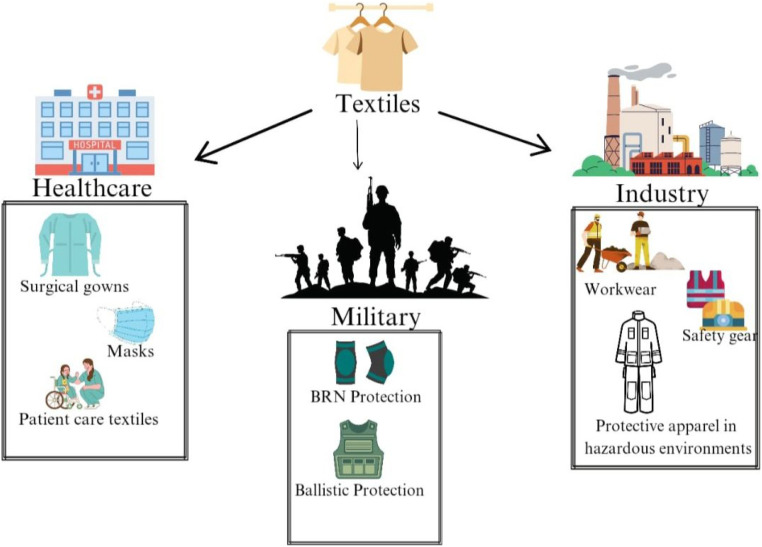
Various uses of textiles in the protective sector.

Fig. 7 shows the significant role of textiles in healthcare, the military, and industry by adjusting to requirements for safety and functionality. Textiles in healthcare are used in surgical gowns, masks, and patient care fabrics, promoting hygiene, infection prevention, and comfort. Specialized textiles for the military offer protection *via* equipment such as bulletproof vests for ballistic safety and materials engineered to defend against biological and radioactive hazards. In industrial environments, textiles are crucial for worker safety, providing robust workwear, helmets, reflective vests, and protective suits for hazardous conditions. This underscores that textiles are not merely fabrics; they are essential for ensuring safety and fulfilling the requirements of many vocations.

Flame retardants are chemical agents formulated to diminish the flammability of materials and impede the propagation of fire. They function by disrupting the combustion process, either by postponing ignition, reducing the rate of heat release, or inhibiting the generation of flammable gases. These effects are accomplished *via* chemical processes that interfere with one or more components of the fire triangle: heat, fuel, and oxygen. Flame retardants significantly enhance fire safety in several industries by modifying thermal deterioration or creating protective barriers. Two major classes of flame retardants are based on halogen or phosphorus. Bromine compounds are usually the most effective among the halogen-containing compounds. The flame-retardant efficacy of halogen compounds is often significantly enhanced when used with antimony compounds, typically Sb_2_O_3_. Phosphorus-based flame retardants are of at least similar significance. Proposals for various materials and end-use applications have been made for members of nearly all classes of organophosphorus compounds. Inorganic phosphorus flame retardants form ammonium phosphates and elemental red phosphorus. The flame-retardant properties of elemental phosphorus may seem unexpected, as its significantly negative oxidation enthalpy underpins the chemiluminescence of the white phosphorus allotrope, which ultimately inspired the term “phosphorus”.^41^ Halogenated flame retardants, previously used extensively, have faced heightened scrutiny, leading to intensified research on halogen-free and phosphorus-based flame retardants specifically. This advancement is additionally ascribed to laws and significant changes in market expectations, as greater emphasis has been placed on developing more sustainable flame retardants.^42^ Phosphorus-based flame retardants have appeared as a significant alternative to halogenated variants. Phosphorus is essential for halogen-free flame-retardancy due to its chemical diversity, several flame-retardant processes, and great efficacy at low concentrations. Phosphorus chemistry is among the oldest branches of chemistry, encompassing the ongoing advancement of novel techniques to enhance the safety and sustainability of chemical processes. Phosphorus-based flame retardants show versatility due to their structural variability, which ranges from inorganic to organic forms, the differing phosphorus content within these molecules, and the presence of phosphorus in various oxidation states, from 0 to +5, leading to distinct flame-retardant mechanisms in both gas and condensed phases. This architectural change makes phosphorus distinctive for the construction of flame retardants with customized property profiles, such as density or glass transition temperatures (*T*_g_), by altering the binding pattern (*e.g.*, from alkyl to phenyl groups).^43^

**Fig. 8:**
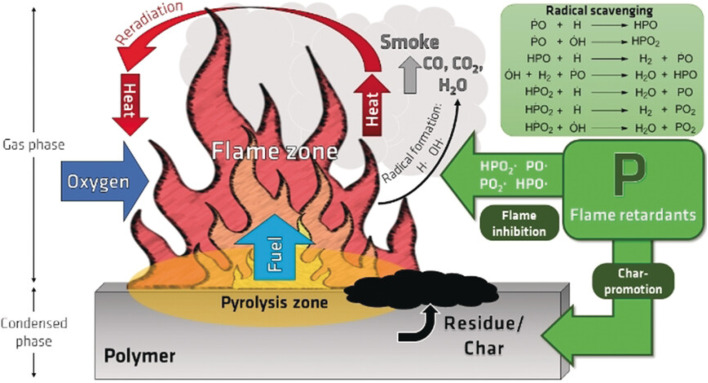
Flaming combustion of polymeric material and the role of phosphorus-based flame retardants. Published under CC-BY-NC License^43^ Copyright 2018, Wiley-VCH Verlag GmbH & Co. KGaA, Weinheim.

Flame retardants impede combustion *via* dual-phase mechanisms. In the gaseous state (see Fig. 8), they emit active phosphorus-containing species, which function as radical scavengers, neutralizing highly reactive radicals such as H˙ and OH˙ in the combustion zone. This interrupts the chain processes essential for supporting combustion. Concurrently, in the condensed phase, flame retardants help the development of a protective char layer on the polymer surface. This character functions as a physical barrier, diminishing the emission of combustible gases (from pyrolysis) and insulating the substrate from heat. Flame retardants efficiently decelerate or inhibit flame propagation by diminishing heat feedback, restricting oxygen availability, and curtailing fuel supply, resulting in the production of less flammable gases (*e.g.*, CO, CO_2_, and H_2_O) and smoke.^44^

### 3.2 Types of flame-retardant materials

Inorganic additives, such as aluminum or magnesium hydroxyl, serve as the primary flame-retardant part in the coating system while also functioning as synergistic co-additives that enhance flame-retardant efficacy. Nonetheless, inorganic fillers often need elevated loadings, which may negatively affect the physical qualities of the coatings. Furthermore, such additives may show incompatibility with the binder system (*e.g.*, UV curable coating), resulting in blooming and other analogous adverse effects. The inorganic metal compounds mostly consisted of titanium, zirconium, and aluminum, which were integrated into the cotton using the sol–gel process. Adding metal compounds enhanced the thermal characteristics of coatings, except the aluminum compound. In comparison to pure cotton, all sol–gel coated fabrics showed an increase in water content of at least 24% upon curing. In the vertical flame tests, the burning rates fell by about 50%, while the char residues rose by around 100–200%. Metal alkoxide coating techniques are regarded as effective in providing fabrics with enhanced flame retardancy. A formulation for a flame-retardant coating consisting of a cross-linked thermosetting polymer based on polyurethane and magnesium hydroxide as a metal hydroxide. This invention delineates the formulation of a coating precursor and a method for applying a flame-retardant layer to a plastic composite plank. The metal hydroxide content of coating matrix (approximately 30%) is said to impart self-extinguishing properties to the substrate.^45^ The layer-by-layer assembly of nanoparticle-based coatings concentrated on the deposition of hybrid organic–inorganic structures composed of anionic clay (montmorillonite or LAPONITE^®^) combined with a cationic polymer (branched polyethyleneimine). The findings, derived from cotton as a substrate, showed enhanced thermal stability and superior flame-retardant characteristics. In pursuit of this research direction, the coating structure has been changed by cutting the organic components to ease the application of a wholly inorganic coating. From a layer-by-layer perspective, the deposition of all-nanoparticle coatings introduces added constraints, rendering it more challenging compared to polyelectrolyte/nanoparticle alternatives. Nanoparticle coatings, consisting of alumina-coated silica (10 nm) and silica nanoparticles (10 or 40 nm), show notable flame-retardant effects on cotton and polyester (PET) materials. These coatings significantly mitigated the melt-dripping phenomenon in PET and prolonged the ignition time during flammability assessments. Excessive stacking, however, reduced the resilience of the covering, hence diminishing overall performance. Horizontal spray deposition appeared as the most effective approach for optimizing the application, guaranteeing uniform coating, and improving thermal barrier qualities during combustion. Furthermore, 50 nm alumina nanoparticles, characterized by their amphoteric properties and pH-adjusted surface charge, were employed to develop multifunctional coatings on cotton. These coatings not only augmented flame retardancy but also bolstered tensile strength and UV resistance, underscoring their multifaceted applicability.^46^ Modified chitosan-based flame retardants offer great potential due to their sustainability, biodegradability, and strong charring properties. Researchers developed two novel bio-based flame retardants by modifying chitosan through phosphorylation and phosphorization reactions. These flame retardants significantly improved the fire resistance of polylactic acid composites. With just 3% of these additives, the materials achieved higher oxygen index values (29% and 27%) and a V-0 rating in vertical combustion tests, demonstrating excellent flame retardancy. However, increasing one type of flame retardant reduced the material's strength. In contrast, the second type maintained better mechanical performance due to improved compatibility with the polymer matrix, balancing fire resistance and structural integrity.^47^

Epoxy resin (EP) is extensively used yet constrained by its flammability, causing the development of environmentally sustainable flame retardants. Chitosan, a prevalent bio-based substance, often employs solvent-intensive, low-yield synthesis techniques that undermine sustainability goals. This work introduces a solvent-free, environmentally friendly method for manufacturing chitosan-based ammonium phytate (PUCS) as a flame retardant for EP. The use of 7.5 wt% PUCS elevated the limiting oxygen index from 22.2% to 32.2% and enhanced the vertical burning rating. Cone calorimeter experiments proven diminished heat release rates, showing superior flame retardancy. The mechanical properties of EP/PUCS composites remained right for engineering applications, providing a sustainable and efficient possibility for flame-retardant EP.^48^

### 3.3 Performance evaluation

**Fig. 9:**
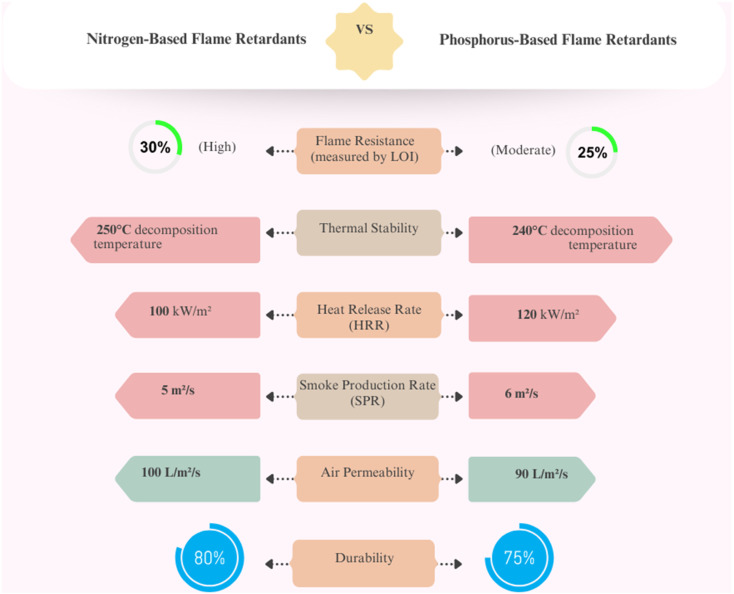
Comparison of flame retardancy performance indicators.

One often-used method to evaluate flammability of fabric numerically is the limiting oxygen index (LOI). It provides a straightforward method to assess the efficiency of flame-retardant treatments over a spectrum of chemical uses since it gauges the lowest concentration of oxygen needed to support combustion. Many traditional flame-retardant treatments for cellulosic fabrics show a linear relationship between LOI values and phosphorus content when nitrogen and phosphorus ratios are kept constant. Tris (aziridinyl) phosphine oxide (APO) deviates from this pattern, however, suggesting special characteristics. Investigating the contribution of nitrogen to improving flame-retardant performance benefits, especially from this method. By exact measurements, one may quantify and investigate the synergistic interplay between nitrogen and phosphorus. Extensive investigations of two chemical systems showed that LOI values may be said as straightforward functions of their nitrogen and phosphorus concentrations. Fascinatingly, the combined effect of nitrogen and phosphorus did not peak at a given ratio, suggesting a more complicated link between the two elements. In vertical flame testing for several materials, an LOI of about 0.26 has been linked to a 5 inch char length; exceptions, including sateen fabric, were noted. Even more than fabric weight, air permeability was found to be a major determinant of the chemical requirements for obtaining efficient flame retardancy. This emphasizes how crucial fabric structure is to decide how effective flame-retardant treatments are^49^ the phenomenon of flame extinction is examined by the LOI test, a prevalent technique in the industry for assessing flame retardancy. The LOI quantifies the minimal oxygen concentration necessary to keep combustion, providing a dependable metric for evaluating a material's resistance to burning. The LOI enables the determination of essential features, including the minimum mass release rate required to extinguish flames and its variation with oxygen levels.^50^ Extinction properties are defined by computing parameters, such as activation energy and reaction rates, based on a simplified single-step chemical reaction during burning. The LOI has been proven to correlate with many flammability characteristics, including heat release and breakdown behavior, as assessed using methods such as thermal analysis and calorimetry. The extinguishment of flames on a solid surface is affected by thermal conduction to the material and the aerodynamics surrounding the flame. Heat transfer is represented by a heat transfer coefficient, as flame radiation near the surface is negligible at the extinction point. The dynamics of airflow, which influence flame stability, are characterized by the ratio of the duration of airflow surrounding the material to the time necessary for chemical reactions. This ratio assesses the rate at which a flame can be extinguished under certain conditions. The critical mass flux for flame extinction is determined using these factors, along with their influence on flow conditions and oxygen concentrations. This method emphasizes the significance of material characteristics, including thermal absorption and chemical reaction velocities, in attaining flame resistance, elucidating the behavior of materials under various fire conditions. Fig. 9 depicts flame retardancy evaluation based on different contributions, including nitrogen and phosphorus relationships *versus* flammability characteristics.

### 3.4 Challenges and trends

**Fig. 10:**
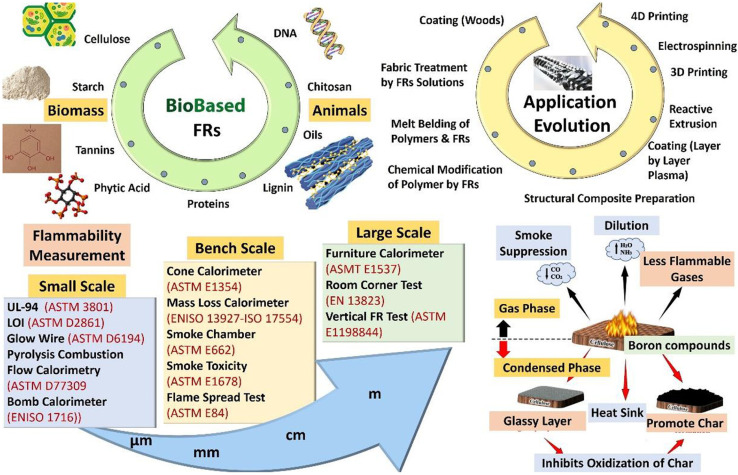
Overview of eco-friendly flame retardants, testing methods, and applications in sustainable materials. Reproduced with permission from ref. 51 Copyright 2024, Sage Publications.

The increasing focus on sustainability has underscored the significance of eco-friendly flame retardants (FRs) as substitutes for conventional, non-renewable, and often hazardous alternatives. These eco-friendly flame retardants, sourced from natural materials such as cellulose, lignin, and chitosan, are essential for improving the fire safety of biobased composites used in vital sectors including automotive, construction, and marine. In contrast to traditional flame retardants, environmentally friendly alternatives are biodegradable, renewable, and show a diminished ecological imprint, consistent with worldwide initiatives to mitigate carbon emissions. In Fig. 10 the graphic illustrates that these flame retardants run *via* mechanisms such as gas-phase dilution and condensed-phase char development to effectively suppress flames. Furthermore, sophisticated testing methodologies of various sizes are essential to assess their efficacy and change them for practical applications. The creation of sustainable flame retardants guarantees fire safety while easing the transition to renewable materials, giving them essential for ecologically responsible advancements in materials science.^51^

Innovations in sustainable and bio-based flame retardants emphasize the development of effective, eco-friendly solutions sourced from natural components such as lignin, chitosan, and phytic acid. This includes the application of nanotechnology to improve fire resistance, specialized coatings for uses, and the incorporation of flame-retardant characteristics into biopolymers and composites. Researchers are investigating plasma treatments and surface modification methods to enhance compatibility and durability. These innovations look to diminish toxicity, improve performance, and bolster global sustainability initiatives across several sectors.

## 4. Antimicrobial coatings

### 4.1 Mechanisms of antimicrobial action

**Fig. 11:**
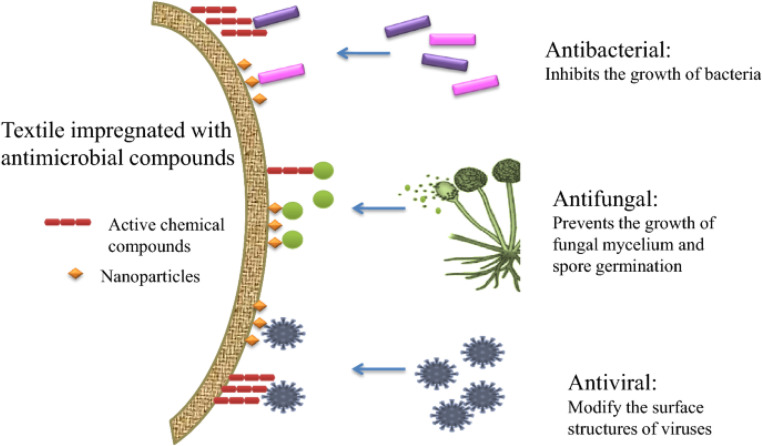
Mechanisms of antimicrobial textiles with active compounds and nanoparticles. Reproduced with permission from ref. 53 Copyright 2021, Springer-Verlag GmbH Germany, part of Springer Nature.

The large surface area and moisture-retaining qualities of textile constructions create a perfect habitat for microorganisms to flourish, which has bad consequences for the fabric and the user. Antimicrobial coatings have been created to solve this by stopping bacterial development, thus improving cleanliness and lowering health hazards.^24^ These coatings use both chemical and physical techniques to include antimicrobial compounds either chemically into textiles or physically onto surfaces.^52^ From healthcare to hygiene items, sportswear, food packaging, air filtration, and water purification systems, antimicrobial textiles have grown indispensable in many different uses. These fabrics are made to either destroy germs or stop their growth, so guaranteeing increased cleanliness and safety. Apart from their antibacterial characteristics, durability is also more important since it guarantees that the functional characteristics resist several washes and extended use without losing efficacy. Moreover, design, color retention, patterns, and other aspects are being combined to satisfy the needs of stylish and multifarious apparel. Reflecting increasing consumer awareness and market demand, many commercial brands today include these elements in their products. Further creativity is driven by developments in synthetic and natural antimicrobial compounds and diagnostic methods to evaluate activity against viruses, fungi, and bacteria. These innovations guarantee that antimicrobial fabrics not only meet practical requirements but also provide modern applications with sustainable, elegant, and high-performance solutions.^53^Fig. 11 shows how fabrics coated with antimicrobial agents, including nanoparticles and active chemical agents, fight different microbes. Antibacterial textiles stop bacterial development; antifungal textiles stop fungal mycelium development and spore germination; and antiviral textiles change the surface structure of viruses to lower their activity. These features provide better safety and cleanliness in many different uses.

Incorporating environmentally safe antimicrobial chemicals as well as metal-based nanoparticles like silver, copper, and zinc oxide has shown amazing advancement in antimicrobial textiles in medical applications. By use of sustainable finishing methods, these materials improve the ability of the fabrics to minimize microbial development, lessen infection rates, and encourage wound healing. The developments underline the important part antimicrobial fabrics play in enhancing healthcare efficiency and safety.^54^

### 4.2 Types of antimicrobial coatings

**Fig. 12:**
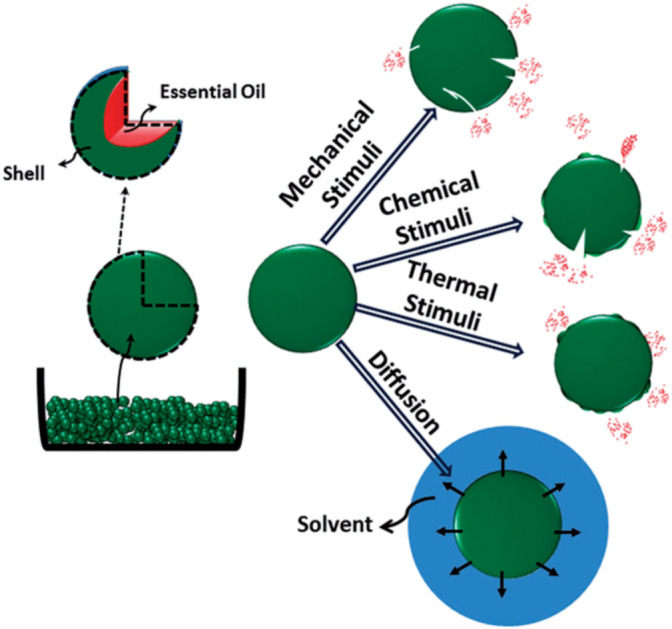
Release mechanisms of essential oils from the micro/nanocapsules. Reproduced with permission from ref. 61 Copyright 2016, Taylor & Francis.

Antimicrobial coatings are classified into natural agents and metal-based agents. Natural agents, such as chitosan and essential oils, originate from sustainable sources and offer environmentally beneficial antibacterial protection.^55,56^ Metal-based treatments, like silver nanoparticles and zinc oxide, show significant efficacy against bacteria and are extensively used for their potent antimicrobial capabilities. Intimate clothing fabrics must have both comfort and antimicrobial qualities. Chitosan-silver finishing on cotton fabric investigates good antibacterial properties against *S. aureus* and *E. coli*.^24,57^ While water vapor transfer, surface friction, and flexibility stayed the same, the final fabric proved enhanced air permeability and hydrophilicity in comparison to the untreated fabric. Within reasonable bounds, chitosan attachment resulted in a slightly increased bending rigidity. Chitosan-silver dual compounding is a potential solution for intimate apparel because it maximizes comfort without sacrificing fabric quality and improves antibacterial efficiency.^58^ Safe and natural linear polysaccharides, chitosan is extensively applied for the multifunctional finishing of textiles. Using core–shell nanoparticles, it has been employed to generate antibacterial cotton fabric with a 99% reduction rate against *S. aureus* where poly(poly(*n*-butyl acrylate)) forms the core and chitosan forms the shell. About 300 nm in size, the nanoparticles were described using FT-IR and ^1^H NMR and then pad-dry-cure covered into cotton fabrics to show outstanding antibacterial efficacy.^59^ Compared to conventional finishes, microencapsulation of essential oils in textiles provides enhanced durability and controlled release. Applied to cotton and polyester cloth, concentrated on microencapsulating citronella oil using gelatin and gum Arabic as shell materials. Fiber type affected the release mechanism; cotton displayed a non-Fickian kinetic model, while polyester displayed Fickian diffusion. These results emphasize the possibilities of essential oil microencapsulation in producing textiles with longer-lasting functional effects.^60^ The processes by which encapsulated substances, such as essential oils, are liberated from microcapsules are depicted in Fig. 12. The release rate, which is triggered by a variety of events, is crucially controlled by the polymeric shell. Mechanical stimulation includes friction or physical pressure that causes the shell to burst, allowing the core material to be released. When external factors like pH shifts, enzymatic activity, or solvents weaken the shell and permit the core to escape, chemical stimulation takes place. While diffusion enables the core material to seep through the shell as external liquids, such as solvents, penetrate the structure, thermal stimuli use heat to melt the shell material, resulting in the core release. Active agents are delivered efficiently and gradually thanks to this controlled release method, which is adapted to applications and environmental circumstances.^61^

Metal-based nanoparticles, especially silver and zinc oxide nanoparticles, show significant potential as antibacterial agents owing to their broad-spectrum efficacy against both Gram-positive and Gram-negative bacteria. Silver nanoparticles are proficient at eradicating germs by compromising their cell membranes, altering metabolic processes, and interfering with DNA, giving them a favored choice for medicinal applications despite ongoing worries about their toxicity. Zinc oxide nanoparticles are successful in eradicating bacteria by comparable methods, offering the advantage of greater safety than silver, albeit with less potency. Both categories of nanoparticles present interesting substitutes for conventional antibiotics, particularly in combating antibiotic-resistant bacteria; yet, their safety, especially with prolonged exposure, continues to be a critical focus of continuing study.^62^

While treating infections is more challenging due to multidrug-resistant bacteria and biofilm formation, noble metal nanoparticles (NMNPs) such as gold, silver, and platinum present a good substitute for conventional antibiotics. By upsetting their cell membranes, attaching to DNA or enzymes, and even producing reactive oxygen species or heat under light, these nanoparticles can kill bacteria. Although they show tremendous promise in combating infections, their toxicity requires careful thought in medical use; hence, it is crucial to make sure their antibacterial force does not compromise safety.^63^ Antibiotic abuses has led to the rise of drug-resistant bacteria, making it harder to treat infections with traditional antibiotics. Metal-based nanoparticles (MNPs), like copper, titanium, and zinc oxide, are being looked at as promising alternatives because they are less likely to cause bacterial resistance and tend to be safer for human cells. This review highlights the antimicrobial potential of gold and silver nanoparticles, which have shown great promise in killing bacteria. It also covers new research on MNPs that can be triggered by light, magnetic fields, or pH changes, making them even more effective. As technology advances, MNPs are expected to be key in overcoming antibiotic resistance and improving treatments for bacterial infections.^64^

**Fig. 13:**
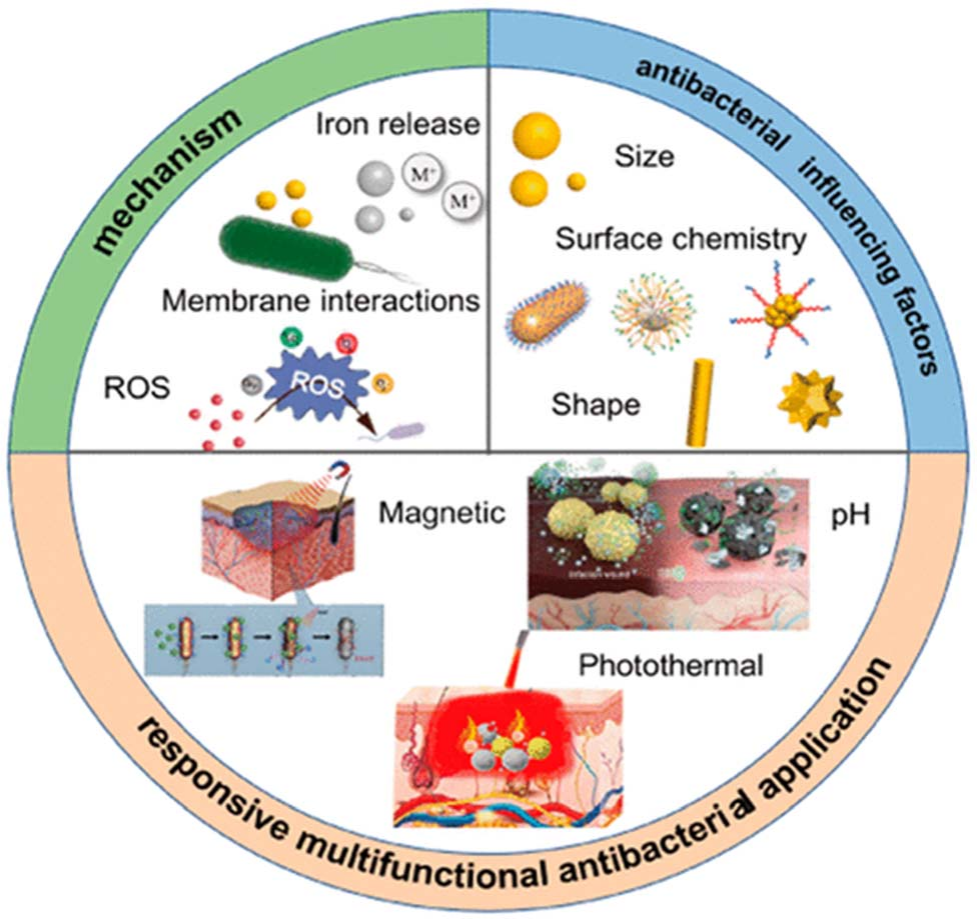
Multifunctional antibacterial mechanisms and applications. Reproduced with permission from ref. 64 Copyright 2024, American Chemical Society.

Fig. 13 shows the processes, influencing elements, and uses of multifarious antibacterial materials. It emphasizes systems such as iron release, ROS formation, and membrane interactions in addition to elements including size, shape, and surface chemistry influencing antibacterial efficacy. Applications include photothermal antibacterial systems, magnetic, pH-responsive, and photochemical systems.

**Fig. 14:**
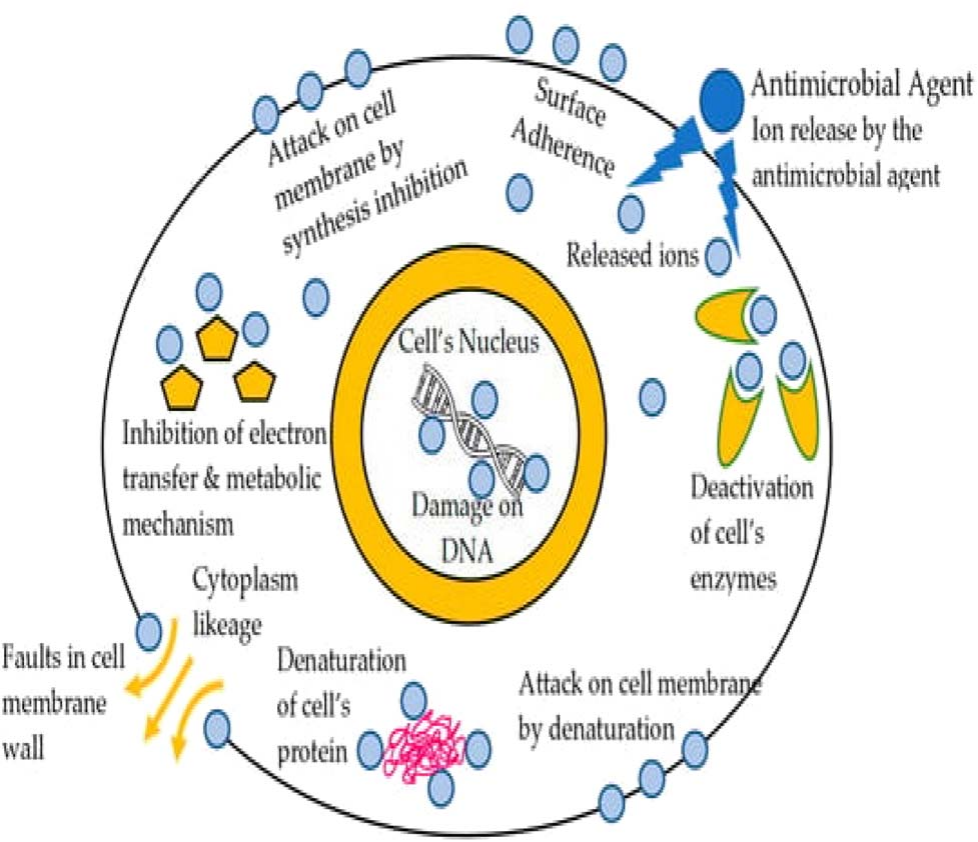
General chemical structure of quaternary ammonium compounds (QACs). Published under CC-BY License^65^ Copyright 2023, MDPI.

Since quaternary ammonium compounds (QACs) (see Fig. 14) are cationic surfactants that efficiently target bacteria, fungi, and certain viruses, they are often used as antimicrobial agents in textiles. To interrupt vital processes and kill microorganisms, their antibacterial effect involves electrostatic interactions between the positively charged nitrogen atom and the negatively charged microbial cell membrane. Although QACs work well, their lack of reactive functional groups prevents them from creating strong chemical connections with fibers, which causes gradual separation and decreased endurance. New developments include sol–gel technology and polymerizable QACs improve durability and wash fastness by forming networks of polymers or covalent connections on textile surfaces. Furthermore, to give dyes their color and antibacterial qualities, QACs have been added, making them suitable for use on both natural and synthetic fabrics, including cotton, wool, polyester, and polyamide.^65^

### 4.3 Durability and wash fastness

Different methods aiming at increasing the durability and adherence of antimicrobial coatings to stop bacterial adhesion and biofilm development are covered in this review. It emphasizes recent developments in surface modifications to improve the long-term performance of antimicrobial surfaces by including hydrophobic, superhydrophobic, and self-cleaning characteristics. To guarantee continuous antibacterial efficacy, the review also covers techniques for strengthening the binding strength of coatings to substrates, like surface functionalization and the use of hybrid materials. It also underlines the need to strike a compromise for useful industrial uses between increasing durability and preserving antibacterial action.^66^ Several techniques are used to increase the durability and adherence of antimicrobial coatings: surfactants, binders, and post-treatment treatments. These methods ensure long-lasting antibacterial activity by helping to improve the stability of the coating and lower leachability. Furthermore, hybrid material compositions and surface functionalization are investigated to increase coating adherence to substrates while preserving their photocatalytic activity. These methods look to solve problems about the endurance and efficiency of photocatalytic antimicrobial coatings in practical settings.^67^ Ensuring the long-term effectiveness and performance in practical uses of antimicrobial fabrics depends critically on standards and methods for evaluating their wash fastness. Usually using ISO 6330 or AATCC 135, these criteria entail assessing the preservation of antimicrobial qualities following several washing cycles. Monitoring bacterial development on the cloth after several washes helps one to evaluate the lifetime of the antimicrobial effect and, hence, its protective qualities. Such testing is crucial to guarantee that antimicrobial textiles keep their safety and utility over time and stay effective.^68^

### 4.4 Biocompatibility and safety

Although medical textiles have great advantages for hospital usage, end users' possible hazards must be carefully evaluated. These hazards include skin irritation or allergic reactions brought on by chemical treatments or finishing chemicals applied in the fabrics, therefore generating negative health consequences. Furthermore, compromising their safety and efficacy could be the deterioration of antibacterial qualities or the release of toxic compounds from textile materials over time. New materials, including those used in implants or medication delivery systems, also need careful study to make sure they do not harm patients over time. Their biocompatibility and durability help to figure out this.^69^ Particularly in recycling and reuse situations, end users of medical textiles run possible hazards, including exposure to dangerous chemicals, germs, or pathogens that could linger on textiles following incorrect treatment or insufficient sterilization. If not properly controlled, the recycling process may undermine the integrity and safety of materials, therefore causing problems including lower mechanical strength, loss of biocompatibility, or the release of harmful compounds from broken-down polymers. Furthermore, the difficulty of preserving the antibacterial qualities and adaptability of medical textiles following recycling could lead to insufficient protection for patients, therefore raising their risk of infections or negative responses. These issues draw attention to the requirement for strict safety procedures and cutting-edge technologies in medical textile reuse and recycling.^70^ One less spoken-about concern of antimicrobial medical textiles is the possibility of inducing antimicrobial resistance. Should these fabrics be worn extensively or their antibacterial qualities fade over time, they could help resistant bacteria proliferate, therefore aggravating future treatments of diseases. The discharge of nanoparticles or chemicals from these materials raises further difficulties as, should they reach out and come into touch with internal tissues or skin, they could be harmful. Although these fabrics can be rather useful, their environmental impact should not be disregarded; if not disposed of correctly, they could leak dangerous chemicals into the surroundings, therefore influencing aquatic life or ecosystems. As we keep developing and using these cutting-edge materials, these are crucial problems that demand more focus.^71^ Although safety and biocompatibility rules for antimicrobial textiles vary depending on the market, their main goals are usually to guarantee that antimicrobial treatments are safe and efficient for human usage. Antimicrobial textiles must follow the REACH (Registration, Evaluation, Authorization, and Restriction of Chemicals) rule in the European Union to guarantee that the chemicals used in textiles are non-toxic and do not endanger the environment. The FDA supervises the safety of textiles used in medical applications in the United States, so antimicrobial treatments must satisfy biocompatibility criteria for devices that come into direct touch with the skin or mucous membranes. Globally, regulations like ISO 10993 for biocompatibility and ASTM F2897 for antimicrobial testing guarantee that these fabrics are safe, durable, and do not negatively affect human health or the environment.^52^ Recent regulatory changes for antimicrobial textiles reflect increased concerns about public health, safety, and environmental effects. Regulations today place a high value on ensuring that antimicrobial agents used in textiles have been properly studied for toxicity and environmental safety, especially as novel materials such as natural polymers and metal-based agents are introduced. The goal is to ensure that these medicines defend against microorganisms without harming people or the environment. Antimicrobial resistance (AMR) is also gaining attention, forcing producers to create effective and long-term treatments. Along with this, there is a drive for more environmentally friendly choices, such as plant-based antimicrobials and smart fabrics, which provide long-term protection without jeopardizing safety. These developing requirements reflect a broader trend of ensuring that antimicrobial textiles are not only functional but also safe, sustainable, and responsible.

## 5. Self-cleaning coatings

### 5.1 Mechanisms of self-cleaning

#### 5.1.1 Photocatalytic cleaning

**Fig. 15:**
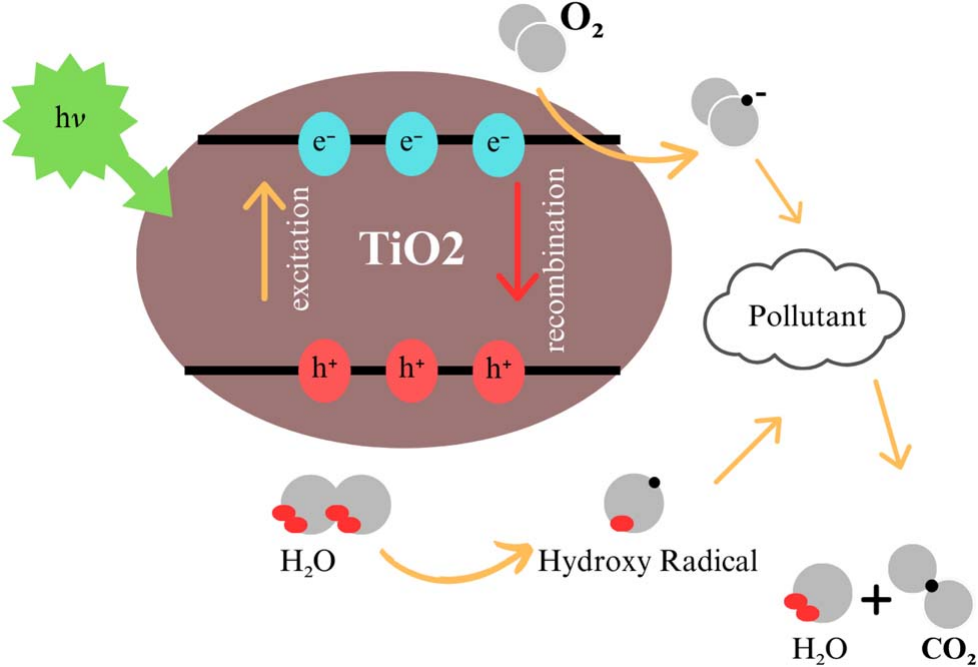
Generation of hydroxyl radicals and reactive oxygen species under light irradiation for pollutant breakdown. Reproduced with permission from ref. 72 Copyright 2021, Springer-Verlag GmbH Germany, part of Springer Nature.

Titanium dioxide (TiO_2_) nanoparticles have revolutionized photocatalysis by providing effective solutions for breaking down organic matter and enabling self-cleaning surfaces (see Fig. 15).^73^ These nanoparticles are highly valued for their chemical stability, non-toxicity, and strong oxidative potential under ultraviolet (UV) light.^74,75^ Upon exposure to UV light with energy equal to or greater than its bandgap (∼3.2 eV for anatase), TiO_2_ generates electron–hole pairs:^73–75^TiO_2_ + *hν* → e^−^ + h^+^

The photogenerated holes (h^+^) oxidize water (H_2_O) or hydroxide ions (OH^−^) to produce hydroxyl radicals (˙OH):^76,77^h^+^ + H_2_O → ˙OH + H^+^

These radicals are highly reactive and degrade organic pollutants into benign compounds like carbon dioxide (CO_2_) and water (H_2_O). Simultaneously, the electrons (e^−^) reduce molecular oxygen to superoxide radicals:^78,79^e^−^ + O_2_ → O_2_^−^

These radicals further take part in breaking down pollutants.

Recent advancements have enhanced the photocatalytic activity of TiO_2_, expanding its utility beyond UV light to the visible spectrum (∼45% of sunlight) through doping with elements like nitrogen, carbon, and metals such as iron and copper.^80–82^ Nanostructuring, including the creation of nanotubes, nanowires, and hierarchical architectures, has further increased the surface area and active sites for photocatalysis.^83,84^ In wastewater treatment, TiO_2_ achieves over 90% degradation of harmful compounds like methylene blue and phenol within an hour of UV exposure.^85,86^ In self-cleaning applications, TiO_2_ coatings decompose organic residues while shifting surface properties from hydrophobic to super hydrophilic (contact angle <5°), ensuring dirt and water removal. TiO_2_-based air purifiers effectively reduce volatile organic compounds (VOCs) like formaldehyde by up to 80%, while their antibacterial properties destroy bacterial membranes and biofilms through ROS generation.^87–89^

#### 5.1.2 Hydrophobic and hydrophilic mechanisms

The self-cleaning capabilities of surfaces have gained significant attention due to their potential applications in environmental sustainability, healthcare, and advanced materials.^90^ These effects are primarily driven by two mechanisms based on surface properties: hydrophobicity (water-repelling) and hydrophilicity (water-attracting). Hydrophobic surfaces show high water contact angles (*θ* > 90°), where water droplets bead up and roll off, carrying away dirt and contaminants.^91,92^ Superhydrophobicity, with contact angles exceeding 150°, is achieved through hierarchical surface structures and low surface energy materials, such as fluorinated silanes or polytetrafluoroethylene (PTFE).^93,94^ The “lotus effect”, inspired by lotus leaves, exemplifies this mechanism, where micro- and nanoscale roughness traps air, minimizing water-surface contact.^95^ This principle is used in self-cleaning glass, reducing maintenance costs by up to 40%, and in treated textiles, enhancing durability and stain resistance.^96^

**Fig. 16:**
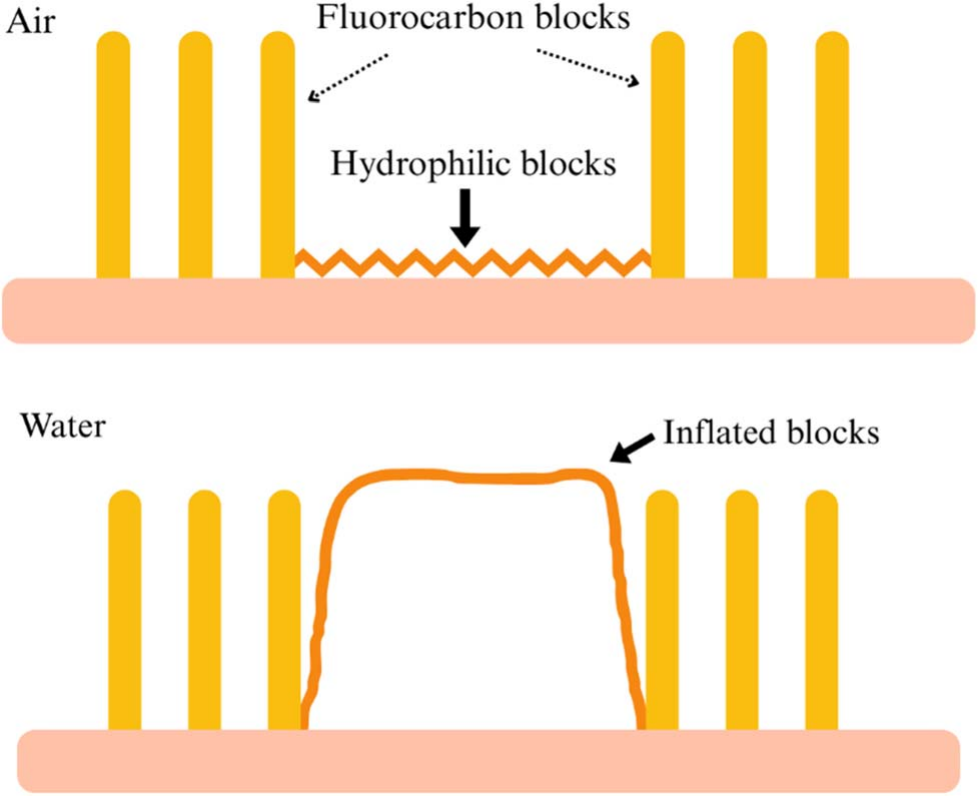
Dynamic surface behavior of textile finishes. Published under CC-BY License^97^ Copyright 2023, MDPI.

As shown in Fig. 16, the interplay between hydrophobic and hydrophilic mechanisms in textile finishing is being controlled by the dynamic behavior of fluorocarbon and hydrophilic blocks. In the top diagram, where the fabric is exposed to air, the fluorocarbon blocks dominate the surface, which are basically hydrophobic in nature. Fluorocarbon chains have low surface energy, which prevents water droplets from spreading or adhering to the fabric. This property is essential for applications like water-repellent clothing, rainwear, or stain-resistant textiles. By orienting outward, these fluorocarbon blocks form a protective layer that resists liquid penetration, ensuring the fabric is still dry and clean even in harsh conditions. The bottom diagram illustrates the fabric's behavior in a water-rich environment, where the hydrophilic blocks come into play. Hydrophilic blocks, which are water-attracting, absorb and interact with water molecules. In this case, they allow the fabric to wick away moisture, enabling it to manage sweat or water effectively. This property is highly desirable in activewear, undergarments, and technical textiles, where moisture transport and breathability are critical for user comfort. When the hydrophilic mechanism is active, the fluorocarbon blocks become less dominant, allowing the fabric to perform optimally in wet conditions.^97^

**Fig. 17:**
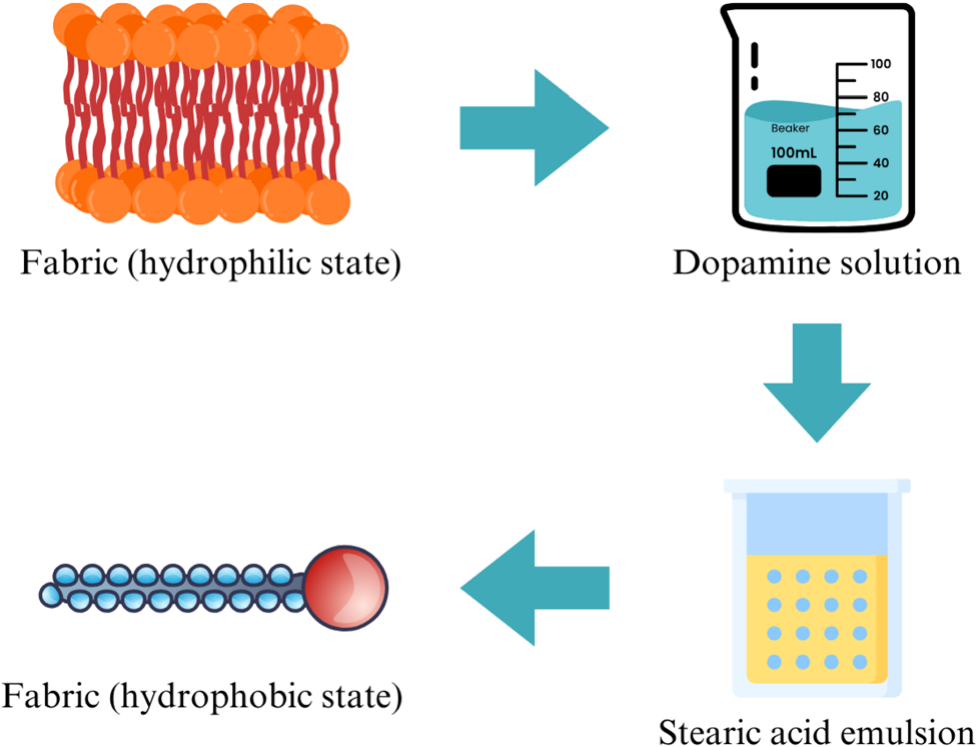
Achieving hydrophobicity through coating by dopamine and stearic acid. Reproduced with permission from ref. 98 Copyright 2019, Royal Society of Chemistry.

This dual-functionality approach is made possible by advanced textile finishing techniques, often involving block copolymers of fluorocarbon blocks. On the other hand, in a different study, a process for transforming textiles from a hydrophilic to a hydrophobic state is achieved through a two-step coating procedure (see Fig. 17). This approach uses the adhesive properties of dopamine and the hydrophobic nature of stearic acid to change the textile surface, creating a durable water-repellent finish. The resulting fabric shows a robust hydrophobic surface, transforming its wetting behavior from hydrophilic to hydrophobic.

### 5.2 Common self-cleaning materials

#### 5.2.1 Photocatalytic agents

Recent advancements have enhanced the photocatalytic efficiency of TiO_2_, addressing its limitation to UV light (∼5% of the solar spectrum). Doping with elements like nitrogen, carbon, or transition metals (*e.g.*, iron and silver) has extended its activity into the visible spectrum, making it more effective under natural sunlight. For instance, nitrogen-doped TiO_2_ can achieve degradation rates comparable to UV-activated TiO_2_ under visible light conditions. Additionally, the development of TiO_2_-based nanostructures such as nanotubes, nanowires, and hierarchical frameworks has increased surface area and light absorption, further boosting efficiency.^83^

Applications of TiO_2_ span diverse fields. In air purification, TiO_2_-coated filters reduce VOCs and harmful gases like formaldehyde by up to 80%. In antimicrobial coatings, TiO_2_ effectively destroys bacterial cell walls through ROS, making it invaluable for hospital sanitation and public health. TiO_2_ is also widely used in self-cleaning surfaces, where it eases the decomposition of organic residues and shifts surface properties to super hydrophilic, enabling easy dirt removal.^99^

Emerging research explores combining TiO_2_ with other advanced oxidation processes (AOPs) like ozonation and UV-C irradiation, which can enhance pollutant degradation. Furthermore, hybrid photocatalysts, such as graphene-TiO_2_ composites, have shown improved charge separation and electron transfer efficiency, resulting in faster and more complete contaminant breakdown. Green synthesis methods for TiO_2_ nanoparticles, using plant extracts or waste products, are also gaining traction to reduce environmental impact. With its versatile applications and ongoing innovations, TiO_2_ continues to lead as a powerful photocatalytic agent, offering scalable and sustainable solutions for addressing global environmental challenges.^100,101^

#### 5.2.2 Superhydrophobic materials

**Fig. 18:**
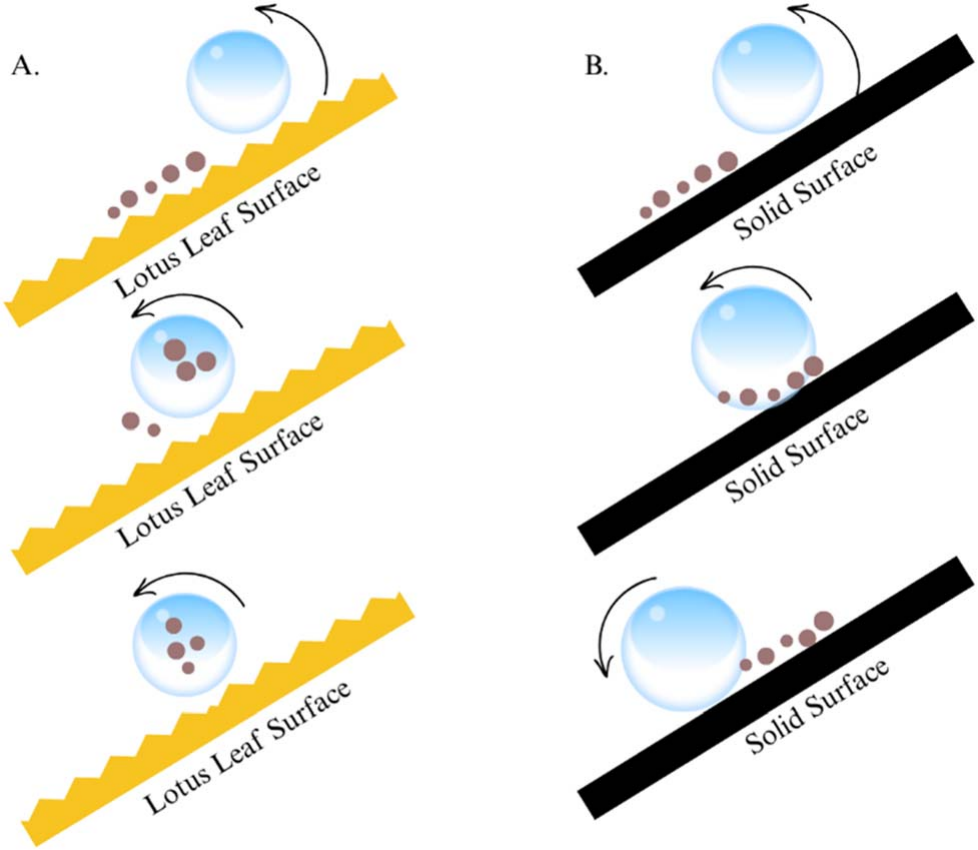
Diagrammatic representation of the “self-cleaning” idea. A drop of water rolling over the surface of a lotus leaf (A) and a smooth, solid surface (B). Reproduced with permission from ref. 102 Copyright 2022, Springer Nature Switzerland AG.

Superhydrophobic materials, inspired by natural phenomena like the lotus leaf effect, show exceptional water-repelling properties with contact angles exceeding 150° (see Fig. 18) and low sliding angles that allow water droplets to roll off effortlessly. Two key strategies for achieving this effect involve the use of silica nanocoatings and fluorinated compounds.^103,104^ Silica nanocoatings play a crucial role in creating the dual roughness required for superhydrophobicity. These coatings, fabricated through methods such as sol–gel synthesis and chemical vapor deposition, introduce micro- and nanostructures that trap air, minimizing contact between water droplets and the surface. Recent advancements include doped silica nanoparticles, such as silica-titanium dioxide composites, which enhance durability and introduce photocatalytic self-cleaning properties and precise lithographic techniques that enable tunable wetting properties.^105^ Surfaces treated with silica nanoparticles often achieve water contact angles up to 170° and sliding angles as low as 2°, with particle sizes in the range of 10–100 nm providing superior performance.

Fluorinated compounds complement silica coatings by reducing surface energy, a fundamental requirement for water repellency. Fluorinated alkyl silanes, such as perfluorooctyltriethoxysilane (PFOTES), chemically bond to silica structures, forming low-energy monolayers that repel water.^106^ Recent research has advanced fluoropolymer-silica hybrids for enhanced durability, developed plasma-assisted fluorination methods to reduce environmental impact, and explored alternatives to traditional perfluorinated compounds due to toxicity concerns. Superhydrophobic surfaces created using fluorinated silanes show water contact angles between 160° and 175°, with modified PFOTES coatings staying effective after over 1000 cycles of abrasion.

The constructive collaboration of silica nanocoatings and fluorinated compounds maximizes superhydrophobicity by integrating structural roughness with low surface energy. These coatings show high thermal and mechanical durability, withstanding temperatures up to 300 °C and keeping performance after repeated stress.^107^ For instance, silica-fluorinated composites achieve water contact angles of 172° and support self-cleaning properties even in corrosive environments. Chemicals like tetraethyl orthosilicate (TEOS) and methyltriethoxysilane (MTES) are commonly used as silica precursors, while perfluorooctyltriethoxysilane (PFOTES) and hexafluorobutyl acrylate serve as fluorinated agents.^108^

### 5.3 Limitations and performance

#### 5.3.1 Durability challenges

Maintaining the self-cleaning properties of materials after repeated washing is a significant challenge, particularly for superhydrophobic and superhydrophobic surfaces. These surfaces rely on delicate hierarchical structures and low-surface-energy coatings to repel water and contaminants, but their performance often degrades due to physical abrasion, chemical interactions, or loss of surface functionality during washing processes.^95^ Abrasion and mechanical wear erode the micro- and nanostructures essential for trapping air and minimizing water adhesion, while detergents and cleaning agents can chemically degrade hydrophobic polymers or fluorinated compounds. Additionally, repeated washing can strip away low-energy coatings, exposing the substrate and reducing water repellency. For super hydrophilic surfaces, contamination by oils or organic residues during washing can interfere with water spreading, diminishing self-cleaning efficiency.

Recent advancements aim to address these challenges by improving coating durability. Nanoengineered coatings with enhanced adhesion, such as silica nanoparticles functionalized with silane coupling agents like (3-aminopropyl) triethoxysilane (APTES), show stronger substrate bonding and keep water contact angles above 150° even after 50 washing cycles.^109,110^ Self-healing coatings, incorporating microcapsules or polymer matrices that release repair agents, restore water-repellent properties after mechanical abrasion or washing, achieving contact angle recovery rates exceeding 90%. Fluorine-free hydrophobic alternatives, such as long-chain alkyl silanes and silicone-based polymers, have been developed to address environmental concerns associated with perfluorinated compounds. These alternatives, combined with nano-patterned structures, resist chemical degradation while keeping contact angles above 160°. Durable micro- and nanostructures, such as titanium dioxide nanowires and graphene oxide composites, show excellent abrasion resistance, keeping self-cleaning properties after prolonged washing cycles.^85^

Hybrid coatings that combine inorganic nanoparticles, such as silica or zinc oxide, with organic polymers like polydimethylsiloxane (PDMS) further enhance durability by using the flexibility of polymers and the wear resistance of inorganic materials.^111^ For instance, silica-PDMS hybrid coatings have kept water contact angles of 165° after 100 washing cycles. Similarly, layered graphene coatings keep self-cleaning properties even after 50 cycles of ultrasonic washing due to their hydrophobic nature and mechanical robustness. Plasma-treated surfaces functionalized with hydrophobic silanes have also shown improved resistance to mechanical and chemical degradation during washing.

#### 5.3.2 Environmental safety

**Fig. 19:**
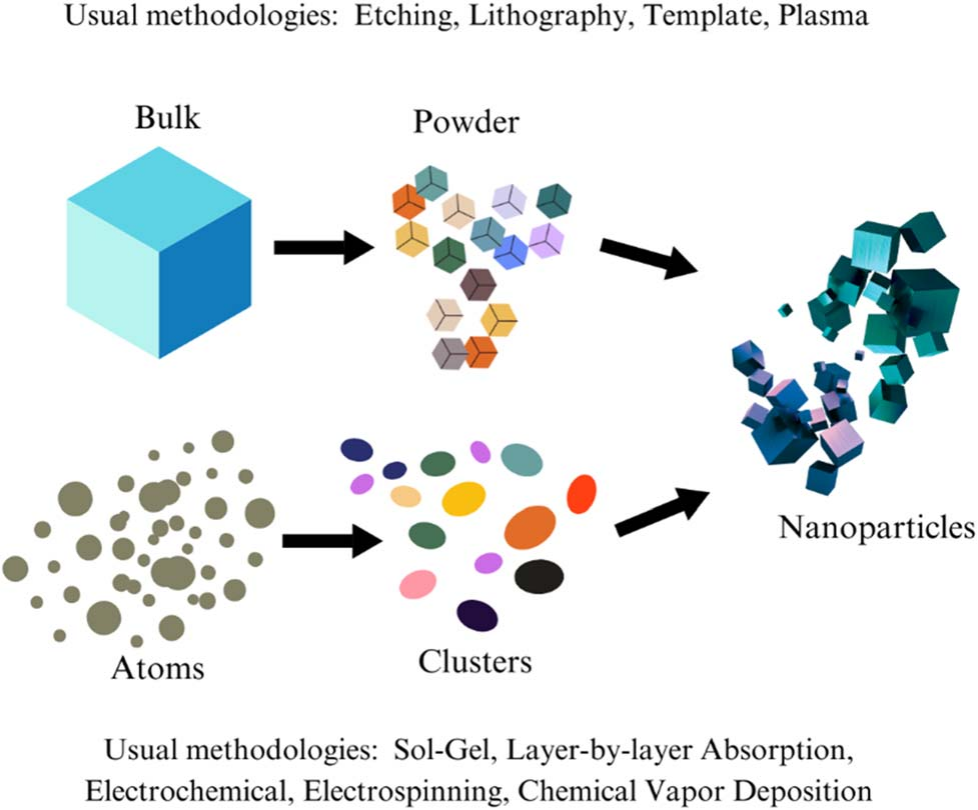
Methodologies that are usually followed to produce nanoparticles. Published under CC-BY License^97^ Copyright 2023, MDPI.

The use of self-cleaning agents, particularly in superhydrophobic and photocatalytic materials, has transformed industries by offering surfaces that repel water, dirt, and pollutants, but their environmental impact is a growing concern.^112,113^ Many self-cleaning agents rely on nanomaterials, fluorinated compounds, and photocatalysts like titanium dioxide (TiO_2_), which pose risks to ecosystems if released into the environment.^114,115^ Nanoparticles such as TiO_2_, zinc oxide (ZnO), and silica (SiO_2_) are crucial for creating micro- and nanostructures or enabling photocatalytic activity (see Fig. 19). Still, their release during manufacturing, use, or degradation can harm aquatic organisms through oxidative stress or bioaccumulation. For instance, TiO_2_ nanoparticles at concentrations as low as 1–10 mg L^−1^ have been linked to reduced growth in algae and crustaceans, while ZnO nanoparticles show potential for bioaccumulation in aquatic food chains. In soil, silica nanoparticles can disrupt microbial activity, affecting nutrient cycles. Fluorinated compounds, such as per- and polyfluoroalkyl substances (PFAS), are another concern due to their persistence, bioaccumulation, and toxicity.^116^ Often called “forever chemicals,” PFAS resist degradation and have been detected in water bodies and soil, sometimes exceeding safe thresholds. Their accumulation in wildlife and humans has been associated with endocrine disruption and reduced fertility. Additionally, photocatalytic agents like TiO_2_ can produce secondary pollutants such as formaldehyde and volatile organic compounds (VOCs) through reactive oxygen species (ROS) generation, while prolonged use can lead to metal ion leaching, further contributing to environmental toxicity.^79^

Recent advancements aim to address these environmental safety concerns. Green nanotechnology has introduced biodegradable alternatives, such as cellulose nanocrystals (CNCs) derived from plant biomass and biopolymer-coated nanoparticles that reduce toxicity while supporting functionality. Non-fluorinated hydrophobic coatings, including silicone-based materials and alkyl ketene dimers (AKDs), are being developed as sustainable replacements for PFAS. Hybrid coatings combining silica nanoparticles with plant-derived hydrophobic agents provide eco-friendly solutions without compromising performance.^117^ Photocatalytic materials are also being improved through doping with metals like silver or gold to enhance efficiency under visible light while reducing harmful by-products. Encapsulation of photocatalysts in protective layers, such as graphene oxide or zeolites, minimizes the release of reactive species and metal ions. Lifecycle assessments (LCAs) are increasingly used to evaluate the environmental footprint of self-cleaning agents, while recycling strategies, such as recovering TiO_2_ nanoparticles from wastewater through filtration, help mitigate environmental release.^118,119^

Studies underscore the importance of these advancements. For example, PFAS-free coatings like those based on AKDs achieve water contact angles of 150°, comparable to traditional fluorinated coatings. Silver-doped TiO_2_ has shown a 30% increase in pollutant degradation efficiency under visible light with 25% fewer ROS by-products, while graphene oxide-encapsulated TiO_2_ reduced metal ion leaching by 70% compared to unmodified materials. Despite these developments, challenges are still in ensuring scalability, cost-effectiveness, and regulatory compliance. Future research is focused on biodegradable nanomaterials, non-toxic hydrophobic agents, and advanced photocatalysts to balance functionality with safety. By integrating lifecycle assessments and robust regulatory frameworks, self-cleaning technologies can evolve to deliver high efficiency with a minimal ecological footprint.^120^

### 5.4 Applications and use cases

#### 5.4.1 Industry applications

**Fig. 20:**
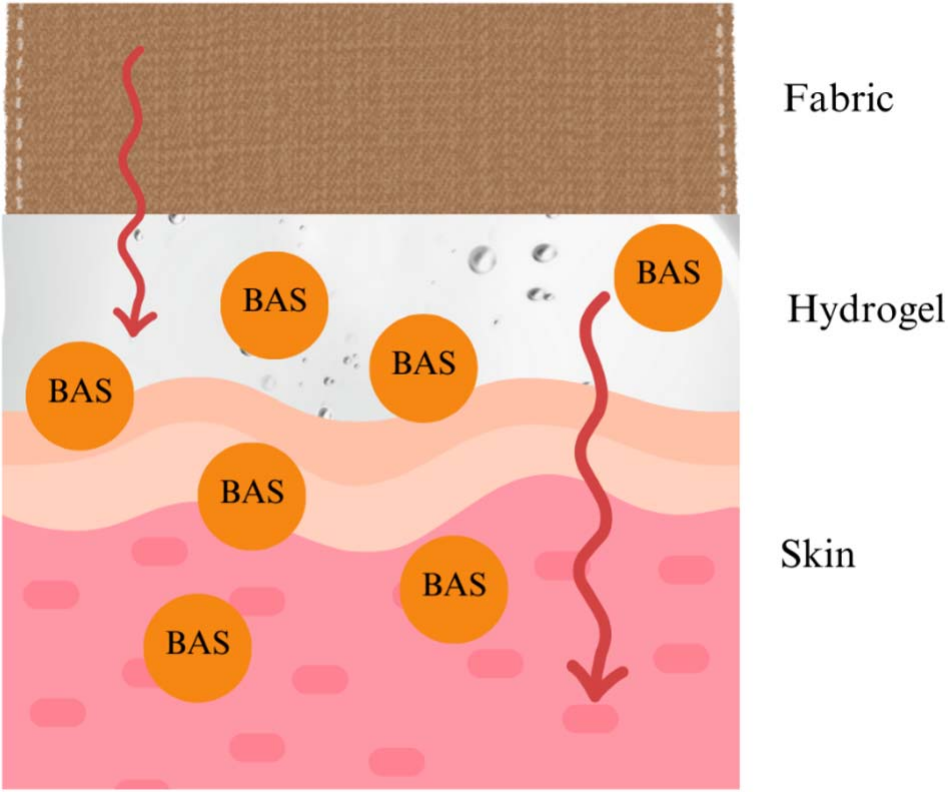
Hydrogel immobilized on a modified cloth releases BAS (biologically active substances). Published under CC-BY License^121^ Copy 2021, MDPI.

**Fig. 21:**
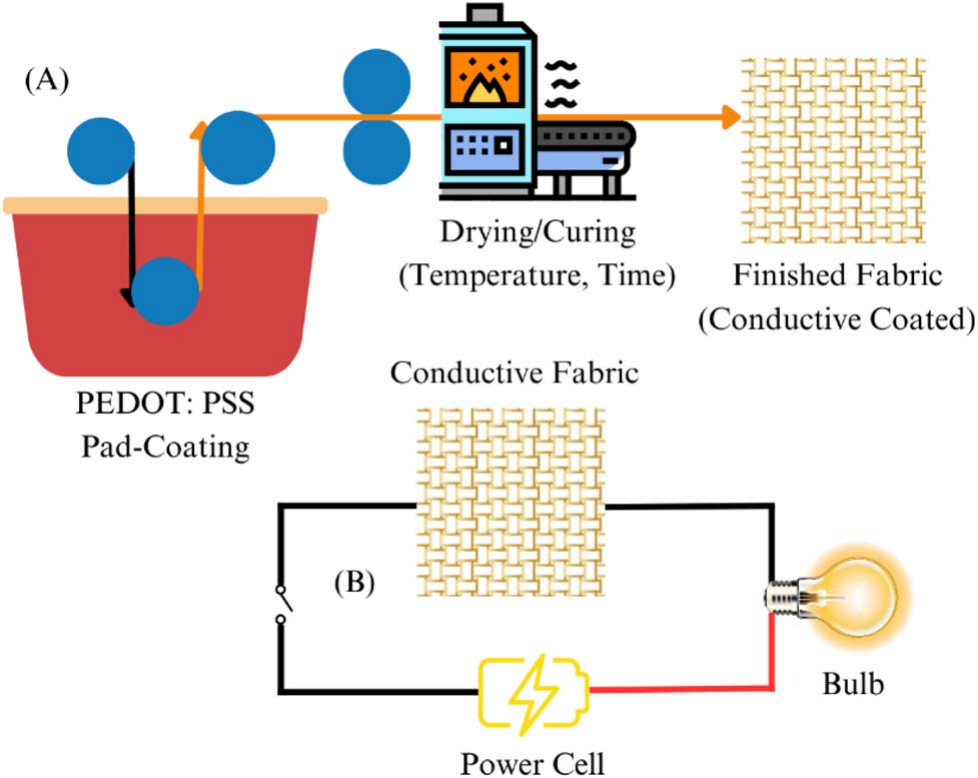
Diagrammatic representation of: (A) poly-3,4-ethylenedioxythiphene:polystyrene sulfonate (PEDOD:PSS) continuous coating process; (B) electrical conductivity testing of pretreated fabric. Published under CC-BY License^123^ Copy 2022, MDPI.

Textiles for healthcare and outdoor applications have advanced significantly, using innovations in materials science, nanotechnology, and functional finishes (see Fig. 21) to meet specific performance demands such as antimicrobial protection, moisture management, UV resistance, and durability. In healthcare, antimicrobial fabrics play a critical role in preventing infections, using agents like silver nanoparticles, zinc oxide (ZnO), and quaternary ammonium compounds (QACs). For instance, silver nanoparticles at concentrations of 10–20 ppm have been shown to reduce bacterial presence by over 99.9% within hours, while ZnO offers long-lasting antibacterial properties with environmental benefits. Barrier textiles, often coated with materials like polytetrafluoroethylene (PTFE) or polyurethane (PU), are used in surgical gowns and masks, with multi-layered nonwoven structures like spunbond-meltblown-spunbond (SMS) composites achieving filtration efficiencies exceeding 95% for particles as small as 0.3 microns. Additionally, smart healthcare textiles incorporating graphene or conductive polymers check health metrics like body temperature and heart rate in real time, while electrospun nanofiber membranes enable controlled drug delivery (see Fig. 20).^122^

Outdoor textiles are designed for durability and comfort in extreme conditions, with advancements including weather resistance, UV protection, and self-cleaning properties. Water-resistant and breathable fabrics treated with durable water repellents (DWR) are widely used, with eco-friendly alternatives like silicone-based finishes and wax emulsions gaining traction. High-performance laminates such as Gore-Tex combine waterproofing, windproofing, and breathability, offering ratings exceeding 20 000 g per m^2^ per day. For UV resistance, agents like titanium dioxide (TiO_2_) and ZnO are incorporated to block harmful radiation, achieving ultraviolet protection factor (UPF) values above 50. Also, achieving conductivity using functional compounds like poly-3,4-ethylenedioxythiphene:polystyrene sulfonate (PEDOD:PSS), thus smart textile functionality (see Fig. 21) can be achieved. Self-cleaning coatings using silica nanoparticles or fluorinated silanes keep cleanliness by repelling water and dirt, while titanium dioxide-based photocatalytic finishes degrade organic stains under sunlight.^124^

Industrially, healthcare textiles are used in antimicrobial scrubs, hospital bed linens, and wound dressings, with nonwoven materials dominating disposable products in a global market exceeding $30 billion in 2023. In outdoor applications, high-performance fabrics are integrated into sportswear, tents, and sleeping bags, using lightweight, breathable materials like Thinsulate and UV-protective clothing with UPF ratings exceeding 50. Military and protective gear use advanced textiles such as Kevlar and Nomex for fire resistance and ballistic protection, while smart textiles with embedded sensors check soldier health and environmental conditions. Supporting data highlights the durability of these innovations, such as antimicrobial fabrics keeping efficacy after 50 washing cycles and self-cleaning coatings maintaining water contact angles above 150° even after extensive washing. Future research focuses on sustainable materials like bio-based polymers and plant-derived nanofibers, along with multi-functional textiles integrating antimicrobial, UV-resistant, and smart features, addressing both performance and environmental demands. These advancements ensure that healthcare and outdoor textiles continue to evolve, meeting industry needs while aligning with sustainability goals.^125^Table 1 depicts certain types of coating performances and other relatable issues.

**Table 1:** Performance metrics for each coating type, including key materials, mechanisms, applications, and measurable performance indicators

Coating type	Key materials	Mechanism and features	Performance metrics	Reference
Flame-retardant (FR)	- Phosphorus-based compounds (ammonium polyphosphate, intumescent coatings)	- Forms a protective char layer, reducing flammability	- Limiting oxygen index (LOI): >25%	Yang *et al.*^126^
	- Metal hydroxides (Mg(OH)_2_, Al(OH)_3_)	- Gas-phase and condensed-phase flame inhibition	- Heat release rate (HRR): reduced by 30–60%	
	- Halogen-free FRs	- Prevents heat and oxygen supply, delaying combustion	- Char residue formation: 2–3× increase	
	- Nanoparticles (TiO_2_, SiO_2_)			
	- Bio-based FRs (chitosan, lignin, DNA-based FRs)			
Antimicrobial (AM)	- Metal-based agents (silver (Ag) nanoparticles, zinc oxide (ZnO), copper (Cu))	- Kills or inhibits bacterial growth	- Bacterial reduction: >99% (ISO 20743)	Bolaños-Cardet *et al.*^127^
	- Natural agents (chitosan, essential oils, tannins)	- Disrupts microbial cell membranes	- Wash durability: effective up to 50 washes	
	- Synthetic agents (quaternary ammonium compounds (QACs), polyhexamethylene biguanide (PHMB))	- Prevents biofilm formation	- Zone of inhibition: ≥2 mm	
Self-cleaning (SC)	- Photocatalytic agents (TiO_2_, ZnO, SiO_2_)	- Photocatalysis: breaks down organic stains and bacteria using light	- Contact angle (superhydrophobicity): >150°	Nomeir B. *et al.*^128^
	- Hydrophobic coatings (silica nanoparticles, fluorinated silanes)	- Superhydrophobicity: water droplets roll off, carrying away dirt	- Dirt removal efficiency: >90%	
	- Graphene oxide & carbon-based nanocoatings	- Superhydrophilicity: water spreads evenly, washing away contaminants	- Photocatalytic degradation: ∼80% pollutant removal in 6 hours	
Multi-functional coatings	- Hybrid nanocomposites (Ag–TiO_2_, SiO_2_–TiO_2_)	- Combines flame-retardant, antimicrobial, and self-cleaning properties	- Retention of functionalities: >80% after 30 washes	
	- Layer-by-layer (LbL) coatings	- Balances durability, sustainability, and performance	- Flame retardancy (LOI): >28%	
	- Bio-based & biodegradable functional coatings	- Optimized for real-world applications	- Bacterial reduction: ∼99% after 50 cycles	
	- Graphene oxide-based multifunctional coatings			He *et al.*^129^
				Liang *et al.*^130^

## 6. Synergistic multi-functional coatings

### 6.1 Integration of multiple functionalities

#### 6.1.1 Combining flame retardant, antimicrobial, and self-cleaning properties

Developing multi-functional coatings that combine flame retardant, antimicrobial, and self-cleaning properties presents a range of challenges despite their immense potential for safety, hygiene, and durability. A key hurdle is the compatibility and stability of diverse chemical components. For example, flame retardants like ammonium polyphosphate (APP) are hydrophilic, while self-cleaning agents such as silica-based hydrophobic coatings require a non-polar environment, leading to phase separation or reduced adhesion. Performance trade-offs further complicate the process, as high loading levels of flame retardants can compromise transparency and flexibility, while antimicrobial agents like zinc oxide (ZnO) nanoparticles can degrade coating material through photocatalytic activity.^131,132^ Scalability also stays a critical challenge, as techniques like layer-by-layer (LbL) assembly, though effective at the laboratory scale, are difficult to implement uniformly in industrial applications.

Environmental and health concerns are increasingly driving the need for sustainable alternatives.^133^ Halogenated flame retardants and silver nanoparticles (AgNPs), while effective, pose risks due to toxicity and bioaccumulation.^134^ Researchers are exploring bio-based solutions like lignin and chitosan to address these issues. Long-term durability is another obstacle, as functionalities like superhydrophobicity in self-cleaning coatings can degrade with wear, while flame retardants and antimicrobial agents may lose effectiveness due to UV exposure or leaching. For instance, coatings with 5 wt% graphene oxide (GO) show a 50% reduction in peak heat release rate (PHRR) but experience a 20% decline in antimicrobial efficacy due to altered surface properties. Similarly, TiO_2_-based coatings with ZnO nanoparticles achieve 90% bacterial reduction but lose 30% of their water-repellent properties after 200 abrasion cycles. To address these challenges, advancing hybrid nanocomposites can be integrated, such components as GO, TiO_2_, and AgNPs for synergistic effects.^134,135^

#### 6.1.2 Materials supporting multi-functionality

Silver-doped titanium dioxide (Ag–TiO_2_) is a versatile agent with significant potential for combining antimicrobial, self-cleaning, and flame-retardant properties, making it ideal for multi-functional material applications in healthcare, construction, and environmental remediation. Its antimicrobial efficacy arises from the synergistic effects of silver ions (Ag^+^), which disrupt microbial cell membranes and inhibit enzymatic functions, and TiO_2_, which generates reactive oxygen species (ROS) under UV or visible light to further damage pathogens. Coatings with Ag–TiO_2_ nanoparticles achieve over 99% bacterial reduction within hours, effectively targeting even antibiotic-resistant strains. Additionally, the photocatalytic properties of TiO_2_, enhanced by silver doping, allow for the breakdown of organic contaminants and enable self-cleaning in low-light conditions.^136^ Ag–TiO_2_ coatings create super hydrophilic surfaces with water contact angles below 5°, ensuring dirt and debris are easily washed away.^137^

Recent research also highlights Ag–TiO_2_'s flame-retardant potential. When incorporated into polymers, it improves thermal stability, catalyzes the formation of protective char layers during combustion, and reduces heat release rates by up to 40%. Beyond safety and hygiene, Ag–TiO_2_ contributes to environmental remediation by degrading pollutants like volatile organic compounds (VOCs) and dyes, achieving over 95% degradation of methylene blue dye under visible light within six hours. However, challenges such as nanoparticle agglomeration and silver leaching persist. To address these, advanced synthesis techniques, like sol–gel methods and plasma-enhanced chemical vapor deposition (PECVD), ensure uniform nanoparticle dispersion, while surface modifications with silica or polymeric layers enhance durability and minimize environmental impact.^138,139^

Supporting innovations include co-doping with metals like copper or zinc to perfect properties and integrating bio-based polymers for sustainability. For instance, Ag–TiO_2_ coatings with 3 wt% silver contents improve photocatalytic efficiency by 20% compared to pure TiO_2_ while maintaining superhydrophilic properties even after 500 abrasion cycles. With ongoing advancements, silver-doped TiO_2_ exemplifies how materials science can deliver integrated, multi-functional solutions for complex challenges across industries.^140,141^

### 6.2 Balancing performance across functions

#### 6.2.1 Trade-offs

Designing multi-functional materials often entails navigating significant trade-offs between critical properties, such as flame retardancy, antimicrobial efficacy, and durability. Balancing these functionalities is a complex challenge, as perfecting one property can inadvertently compromise another. For instance, achieving high flame retardancy typically requires substantial loading of additives like ammonium polyphosphate (APP) or aluminum hydroxide, which can weaken the mechanical properties of the material, reducing its flexibility or impact resistance. Similarly, integrating antimicrobial agents such as silver nanoparticles (AgNPs) or zinc oxide (ZnO) into coatings can enhance hygienic performance but may decrease long-term durability due to ion leaching or oxidative degradation under environmental stressors.^142,143^

One common compromise arises between flame retardancy and durability. Phosphorus-based flame retardants, while effective, often create porous char layers during combustion, which can degrade structural integrity over time. For example, adding 20 wt% APP to a polymer matrix can reduce the peak heat release rate (PHRR) by up to 60%, yet also diminish tensile strength by 30%, limiting its application in load-bearing materials. Similarly, antimicrobial agents like AgNPs enhance bacterial reduction by over 99% within hours but can accelerate the degradation of organic polymer matrices through photocatalytic reactions when exposed to UV light.^80,86^

Another critical trade-off is seen in the integration of self-cleaning functionalities with mechanical durability. Superhydrophobic coatings, which rely on low surface energy materials like polytetrafluoroethylene (PTFE) or silica nanoparticles, often lose their water-repellent properties after abrasion. For instance, coatings with water contact angles above 150° may experience a 40% reduction in performance after 200 abrasion cycles, making them less effective in harsh environments. By using hybrid systems and creative material designs, recent developments have tried to lessen these trade-offs. For instance, while preserving structural integrity, graphene oxide (GO) and TiO_2_ nanoparticles combine to improve flame retardancy and photocatalytic self-cleaning capabilities. Coatings with 5 wt% graphene oxides show a 50% reduction in PHRR and retain superhydrophobicity even after 500 wear cycles. Similarly, encapsulating antimicrobial agents within silica or polymeric shells reduces leaching and extends longevity without compromising bacterial efficacy.^104,106^

The use of co-doped materials, such as TiO_2_ doped with both silver and copper, has also been explored to balance antimicrobial and photocatalytic properties while minimizing environmental degradation. Computational models and machine learning further aid in predicting and improving trade-offs, enabling the design of multi-functional materials that achieve a harmonious balance between performance and durability. Despite these advancements, understanding and addressing the intricate trade-offs between functionalities is still a cornerstone of multi-functional material research.^141,143^

#### 6.2.2 Stability of multi-functional coatings

Ensuring stability and compatibility in multi-functional coatings stays a significant challenge in material science, as these coatings often integrate diverse chemical components to deliver properties like flame retardancy, antimicrobial activity, and self-cleaning functionality. The inherent differences in the chemical and physical behavior of these components can lead to phase separation, poor adhesion, or reduced overall performance. For instance, flame retardants such as ammonium polyphosphate (APP) and aluminum hydroxide are hydrophilic, while self-cleaning agents like silica-based superhydrophobic coatings or fluoropolymers require hydrophobic environments. This incompatibility can result in weak interfaces and compromised structural integrity, particularly under thermal or mechanical stress.^144,145^

One major issue is the stabilization of nanoparticles like silver (Ag) or titanium dioxide (TiO_2_), which are commonly used in antimicrobial and self-cleaning applications. Silver nanoparticles, while highly effective against microbes, are prone to agglomeration due to their high surface energy, leading to uneven distribution and reduced efficacy. Similarly, TiO_2_ nanoparticles, which rely on photocatalytic activity, may degrade other components in the coating under UV light, undermining durability and performance. For example, coatings with 2 wt% Ag or TiO_2_ nanoparticles have proved a 30% reduction in antimicrobial efficacy due to agglomeration and a 25% decrease in mechanical strength after prolonged UV exposure.^146,147^

The interaction between active components can further worsen stability issues. In some cases, antimicrobial agents like zinc oxide (ZnO) can catalyze the breakdown of flame-retardant additives, reducing their effectiveness. Similarly, phosphorus-based flame retardants may react with metal oxides, altering the chemical structure and leading to reduced flame retardancy or the release of volatile by-products. Studies show that coatings with dual functionalities—such as those combining 10 wt% APP with 3 wt% Ag–TiO_2_ nanoparticles—show a 20% reduction in flame retardancy compared to single-function formulations due to such chemical interactions.^132^

To address these challenges, researchers have explored advanced methods to enhance stability and compatibility. Surface functionalization of nanoparticles, such as grafting silica or polymer layers onto Ag or TiO_2_, has been shown to improve dispersion and reduce adverse interactions. For example, silica-coated Ag nanoparticles support over 90% antimicrobial activity after 200 hours of UV exposure and show improved compatibility with hydrophobic matrices.^148^ Similarly, the use of compatibilizers like organosilanes or phosphonate-based coupling agents improves adhesion and stability between hydrophilic and hydrophobic components.^92,149^

Recent innovations also include hybrid nanocomposites, where materials like graphene oxide (GO) serve as a matrix to stabilize nanoparticles and enhance their synergistic properties. Coatings with 5 wt% GO and 3 wt% Ag–TiO_2_ have proved a 40% increase in mechanical strength and a 50% reduction in agglomeration compared to conventional systems. Computational tools and molecular dynamics simulations are also being used to predict interactions between components, enabling optimized formulations with enhanced stability.^85,134^

### 6.3 Case studies

#### 6.3.1 Successful multi-functional applications

The successful application of multi-functional materials in medical, military, and consumer textiles highlights the transformative potential of functional coatings and integrated functionalities. In medical textiles, the demand for antimicrobial, self-cleaning, and biocompatible fabrics has led to innovations such as silver nanoparticle (AgNP) coated surgical gowns and wound dressings. These textiles effectively combat bacterial infections, with studies showing over 99% reduction in pathogens like *E. coli* and *Staphylococcus aureus* within hours of contact. Additionally, incorporating titanium dioxide (TiO_2_) nanoparticles enhances photocatalytic activity, enabling self-cleaning under UV or visible light, crucial for keeping sterile environments. For instance, textiles coated with a combination of AgNPs and TiO_2_ nanoparticles prove sustained antimicrobial activity and self-cleaning efficacy after 50 wash cycles, addressing durability concerns in healthcare settings.^150,151^

In military applications, textiles are designed to offer flame retardancy, chemical resistance, and antimicrobial properties to protect personnel in extreme conditions. Flame-retardant uniforms, for example, integrate phosphorus-based additives like ammonium polyphosphate (APP) into high-performance aramid fibers, reducing the peak heat release rate (PHRR) by up to 60%. Simultaneously, antimicrobial agents such as zinc oxide (ZnO), or copper nanoparticles ensure hygiene in prolonged field operations by preventing microbial growth and odors. Recent advancements include hybrid coatings that combine silica-based superhydrophobic layers with APP and ZnO, achieving multi-functionality without compromising mechanical strength. These textiles support water repellency and antimicrobial efficacy after exposure to harsh environments and abrasion, meeting the rigorous demands of military use.^152,153^

In the consumer textile sector, multi-functional fabrics cater to growing consumer preferences for convenience, safety, and sustainability. Self-cleaning fabrics treated with fluorinated silanes or silica nanoparticles achieve water contact angles above 150°, ensuring excellent hydrophobicity. Combined with photocatalytic agents like TiO_2_, these fabrics can degrade organic stains and pollutants, reducing the need for frequent washing. Antimicrobial finishes, incorporating natural compounds like chitosan or synthetic agents like silver ions, are increasingly integrated into everyday clothing to enhance hygiene. For example, consumer textiles with AgNP coatings keep antimicrobial activity above 90% after 30 wash cycles, proving their durability and practicality.^154,155^

Emerging trends include the use of graphene oxide (GO) and carbon nanotubes (CNTs) to achieve enhanced mechanical properties alongside multi-functionality.^122^ In military textiles, GO-reinforced fabrics improve ballistic resistance while supporting flame retardancy and antimicrobial properties. Similarly, in medical textiles, GO-based coatings combined with AgNPs show dual antibacterial and antiviral activity, addressing broader healthcare challenges. In consumer textiles, CNT-infused coatings improve electrical conductivity, enabling the integration of smart features like temperature regulation and activity monitoring, further expanding the functionality of fabrics.^107,156^

#### 6.3.2 Lessons from real-world applications

Real-world applications of multi-functional coatings and materials have provided valuable insights into the challenges and successes of integrating diverse properties such as flame retardancy, antimicrobial activity, and self-cleaning functions into practical products.^157^ In industries such as healthcare, textiles, and construction, the adoption of these advanced materials has often highlighted critical considerations around performance, scalability, and long-term durability.^158,159^

In the healthcare sector, for instance, silver nanoparticle (AgNP)-coated fabrics used in surgical gowns and wound dressings have proved remarkable effectiveness in reducing bacterial growth.^160^ However, lessons learned from industrial-scale production reveal that while AgNPs show significant antimicrobial activity, they can experience challenges in consistency and stability across batches. Over time, the leaching of silver ions from the coating—particularly under repetitive washing or prolonged exposure to moisture—can reduce the antimicrobial effect.^161^ To address this, manufacturers have increasingly adopted silica or polymeric encapsulation of AgNPs, which not only slows the leaching process but also improves the uniformity and long-term stability of antimicrobial properties.^162,163^ For example, studies on AgNP encapsulated in silica show that the antimicrobial efficacy of coatings can be kept up to 50 wash cycles, compared to 15–20 cycles in unencapsulated formulations.^164,165^

In the military sector, the need for multi-functional textiles that are flame-retardant, durable, and lightweight has driven the development of advanced aramid-based fabrics treated with phosphorus-based flame retardants, like ammonium polyphosphate (APP).^166^ While these coatings are effective at reducing flame spread and heat release, the challenge lies in keeping their durability under field conditions, where textiles are subjected to abrasion, UV exposure, and chemical exposure. In practical applications, adding flame retardants can cause a decrease in the mechanical strength of the fabrics, potentially compromising their utility in combat situations. Recent innovations in hybrid coatings, which combine flame retardants like APP with graphene oxide (GO), have improved the balance between flame resistance and mechanical durability. For example, aramid fabrics treated with 10 wt% APP and 3 wt% have shown a 30% improvement in tensile strength, alongside a 50% reduction in peak heat release rate (PHRR), illustrating how combining materials can enhance overall performance.^166,167^

**Fig. 22:**
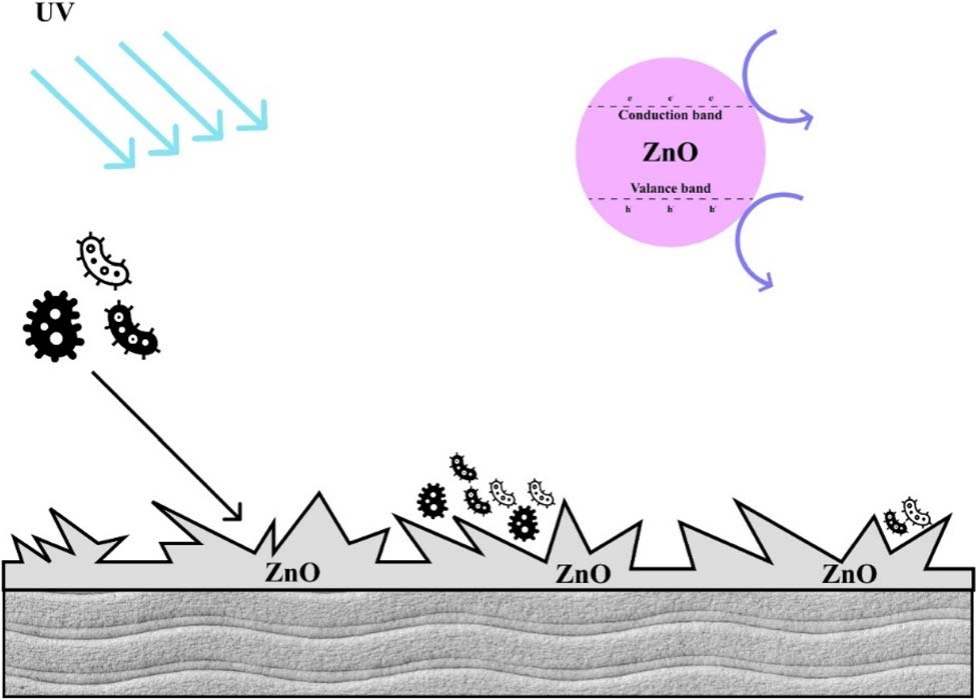
Soil release finished using ZnO nanoparticle. Published under CC-BY License^168^ Copyright 2025, Springer Nature.

In the consumer textile market, multi-functional fabrics are gaining traction for their self-cleaning, soil-release properties (see Fig. 22) and water-repellent properties. These textiles, often treated with superhydrophobic coatings made from fluorinated silanes or silica nanoparticles, offer practical benefits in reducing the need for frequent washing and increasing longevity. However, real-world challenges with these coatings involve their susceptibility to wear and tear, especially when exposed to mechanical stress or repeated washing.^169^ Consumer insights from product durability testing have led to the development of more robust coatings that combine fluoropolymers with photocatalytic agents like titanium dioxide (TiO_2_), which not only provide long-lasting hydrophobicity but also help break down organic stains over time. A notable development is the use of TiO_2_-embedded self-cleaning coatings that are still effective for up to 100 wash cycles, compared to the usual 20–30 cycles seen with older formulations.^170,171^

**Fig. 23:**
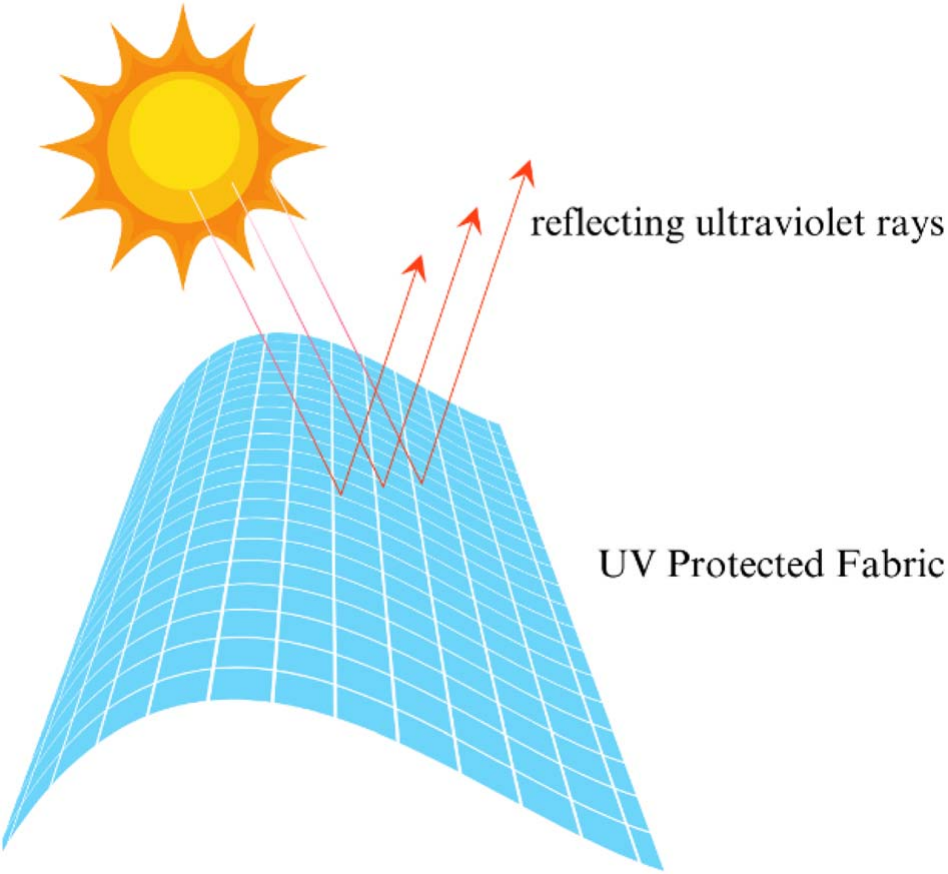
How UV-protective fabrics work. Published under CC-BY License^172^ Copyright 2024, Wiley Periodicals LLC on behalf of Society of Plastics Engineers.

One critical takeaway from these industries is the importance of material compatibility and stability when designing multi-functional coatings. For example, coatings that combine antimicrobial agents with flame retardants in building materials have had mixed results, as the high loading of flame retardants can reduce the effectiveness of antimicrobial compounds due to chemical interactions. To mitigate these effects, recent developments have focused on incorporating nanomaterials like zinc oxide (ZnO) (see Fig. 22) or graphene oxide (GO) in ways that improve compatibility without compromising functionality. ZnO has shown promise in achieving both antimicrobial and UV-blocking properties in coatings for building materials (see Fig. 23), extending their lifespan and functionality in outdoor applications.^173,174^

Another lesson from these industries is the importance of scalability and cost-effectiveness in manufacturing multi-functional materials. While laboratory-scale coatings show impressive results, translating these innovations to large-scale production often reveals challenges related to uniformity, cost, and efficiency. For instance, the process of incorporating nanoparticles like AgNPs or TiO_2_ into textiles often requires precision in dispersion techniques, such as sol–gel or dip-coating methods. In practice, ensuring that nanoparticles are evenly distributed across large fabric surfaces stays a significant hurdle, changing the consistency and cost of mass production. Hybrid approaches, such as using simpler, more cost-effective alternatives like carbon-based nanomaterials or bio-based flame retardants, have gained popularity in streamlining manufacturing while keeping performance.^175,176^ Multifunctional coatings can solve a variety of problems in a range of applications, including greater cleanliness and lower maintenance through antibacterial and self-cleaning properties in addition to increased safety through flame resistance.^177^ However, in order to balance the qualities without sacrificing any one capability, precise material selection and formulation techniques are needed to achieve optimal performance. In order to overcome issues with the stability and compatibility of these integrated systems and eventually provide more sustainable and efficient protective textile solutions, ongoing research is crucial.^178^

A number of important research directions are proposed in response to the question of the future of multifunctional coatings and next-generation textile technologies. First, it is advised that sustainable and bio-based materials be developed for environmentally friendly coatings that yet function well. To guarantee their broad use in a variety of industries, attention should also be paid to improving the scalability and affordability of novel coatings.^179^ Furthermore, to aid in the development of new functionalities, interdisciplinary techniques that combine textile engineering, chemistry, and materials science are encouraged. It is also thought to be essential to conduct more research on the long-term safety and durability of these multipurpose coatings in practical applications. In order to close the gap between research and real-world application and propel the development of next-generation textile technologies, cooperation between academia and industry is highlighted as a final strategy.^180,181^

Table 2 discusses how the chemical and physical interactions between different functional agents occurred in different studies, given that some functional agent combinations can lead to degradation issues, such as photocatalytic agents (*e.g.*, TiO_2_, ZnO) breaking down flame-retardant coatings under UV exposure. To prevent incompatibility, strategies like layering techniques and surface modifications can be employed to maintain coating stability and performance. Additionally, certain materials, such as SiO_2_ and chitosan, offer inherent multi-functionality, providing both flame-retardant and antimicrobial properties. This reduces the need for multiple additives, enhancing the overall efficiency and durability of functional coatings.

**Table 2:** Chemical and physical interactions between different functional agents in textile coatings

Functional agents	Chemical interactions	Physical interactions	Reference
Flame-retardants (FR) + antimicrobial agents (AM)	- Some phosphorus-based FRs may react with metal-based antimicrobials (*e.g.*, silver, copper), affecting their stability	- Metal-based antimicrobials (Ag, Cu) may alter the char formation in FR-treated textiles	Yang *et al.*^126^
	- Halogenated FRs can release reactive species that degrade antimicrobial coatings	- FR coatings can form a barrier that affects the diffusion of antimicrobial agents	Ghosh *et al.*^148^
Flame-retardants (FR) + self-cleaning agents (SC)	- Photocatalytic agents (TiO_2_, ZnO) in SC coatings can degrade some FR coatings under UV exposure	- Hydrophobic self-cleaning coatings may alter flame retardancy by changing the heat transfer and oxygen exposure	Yang *et al.*^126^
			Nomeir *et al.*^128^
Antimicrobial agents (AM) + self-cleaning agents (SC)	- Silver (Ag) and zinc oxide (ZnO) antimicrobials are also photocatalytic, potentially enhancing self-cleaning effects	- Self-cleaning layers may act as a physical barrier, slowing the release of antimicrobial agents	Ghosh *et al.*^168^

## 7. Environmental and safety considerations

### 7.1 Environmental impact

#### 7.1.1 Eco-toxicity of coating agents

Concerns over the eco-toxicity of coating materials, especially those used in multi-functional applications like flame retardancy, antimicrobial protection, and self-cleaning, are becoming increasingly important as their widespread use may result in environmental contamination. Many of the chemicals and compounds incorporated into these coatings, such as silver nanoparticles (AgNPs), titanium dioxide (TiO_2_), and certain phosphorus-based flame retardants, have raised concerns about their potentially harmful impact on ecosystems when they leach into water bodies, soil, or air. For example, silver nanoparticles, while effective as antimicrobial agents, are highly mobile in aquatic environments and can bioaccumulate in organisms like fish, leading to toxicity. Studies have shown that AgNPs can disrupt the gill and liver function of aquatic organisms, with concentrations as low as 1 mg L^−1^ causing significant changes in behavior and mortality rates. Similarly, their leaching from textiles and coatings into wastewater has led to concerns about the contamination of water sources, posing risks to aquatic biodiversity.^182,183^

Titanium dioxide (TiO_2_), often used for its photocatalytic properties in self-cleaning coatings, is another compound that presents eco-toxicity concerns, particularly in its nanoparticulate form. While TiO_2_ is generally considered less toxic than other nanoparticles, its widespread use in consumer products has raised alarms due to its persistence in the environment. Under UV exposure, TiO_2_ can generate reactive oxygen species (ROS), which may cause oxidative stress in aquatic organisms and lead to DNA damage. Studies show that TiO_2_ nanoparticles, when released into the environment, can accumulate in the soil and water, affecting microorganisms that are vital for nutrient cycling and ecosystem health. Furthermore, research suggests that the photocatalytic action of TiO_2_, while beneficial for self-cleaning purposes, may inadvertently degrade natural organic compounds in ecosystems, causing unintended environmental impacts.^184,185^

Phosphorus-based flame retardants, like ammonium polyphosphate (APP) and aluminum hydroxide, are also a cause for concern due to their potential to leach into water systems over time. While these compounds are effective in reducing flammability, they have been linked to increased toxicity in aquatic life, including disruption of reproduction and growth in fish and amphibians. APP can break down into more toxic by-products under environmental conditions, further worsening its environmental impact. Research has shown that flame retardants, when released from materials like fabrics and building materials, can lead to bioaccumulation in the food chain, affecting both marine and terrestrial ecosystems.^186–188^

The increasing focus on the eco-toxicity of coating materials has prompted efforts to develop more sustainable alternatives. For example, bio-based flame retardants derived from natural sources, such as plant oils and polysaccharides, are being explored to reduce the environmental footprint of coatings. Similarly, nanomaterials like graphene oxide (GO) are being considered for their lower toxicity profiles and enhanced compatibility with natural ecosystems. GO-based coatings have shown promise in providing antimicrobial and flame-retardant properties without the same level of toxicity as traditional nanoparticles like AgNPs or TiO_2_. Moreover, researchers are focusing on improving the stability and biodegradability of these materials to prevent long-term environmental accumulation. For example, some studies have proved that organic-based flame retardants, when incorporated into coatings, can be less persistent in the environment, breaking down into non-toxic compounds over time.^189,190^

The drive towards more eco-friendly multi-functional coatings is leading to the development of “green” alternatives that provide the same or better performance while reducing environmental harm. These include coatings made from renewable resources such as chitosan (a biopolymer derived from chitin) or non-toxic, plant-based antimicrobial agents. Chitosan, for example, has shown antimicrobial activity comparable to silver-based coatings but with much lower environmental toxicity. Similarly, bio-inspired coatings, such as those based on natural waxes and oils, are being engineered to mimic self-cleaning and hydrophobic properties while being more easily biodegradable. Despite these advancements, balancing the multifunctionality of coatings with their eco-toxicity stays a key challenge.^191,192^

#### 7.1.2 Biodegradability

**Fig. 24:**
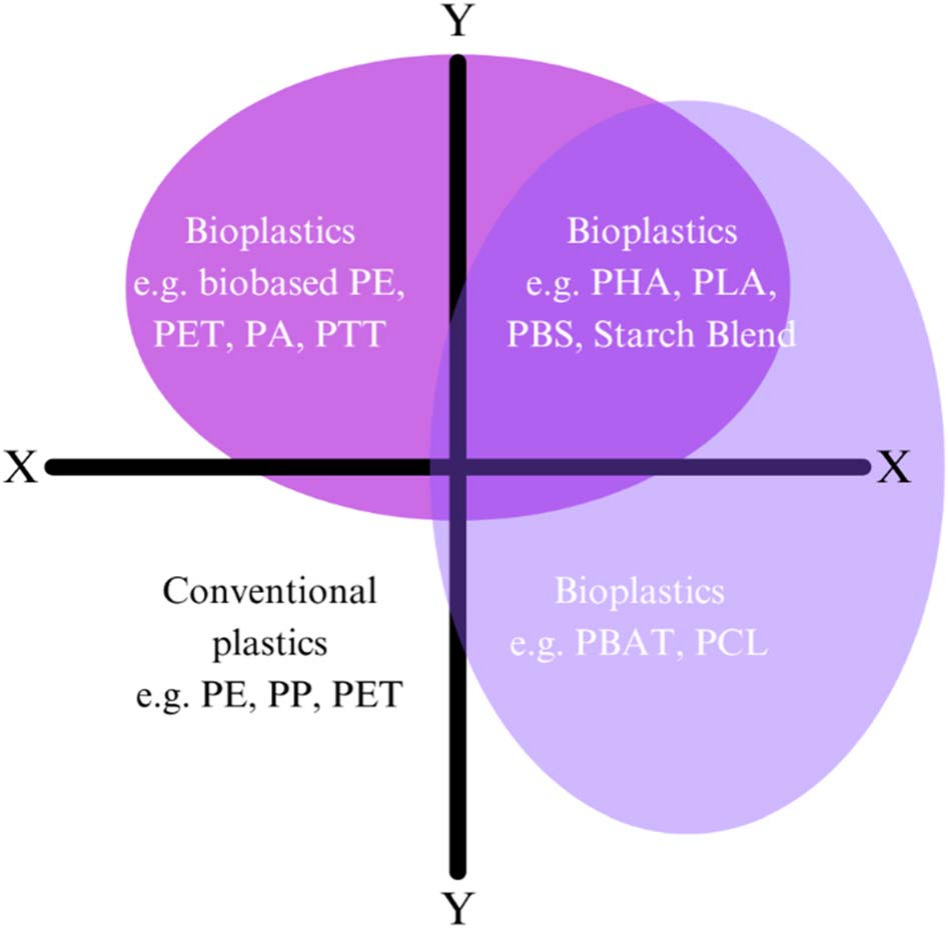
Bioplastics and conventional plastics in Venn diagram.

Recent advancements in biodegradable and non-toxic coatings have made significant strides toward reducing environmental impact while supporting performance in various applications. Traditionally, many coatings relied on synthetic polymers and chemical additives, which often led to environmental pollution due to their resistance to natural degradation. However, increasing concerns about environmental sustainability and eco-toxicity have driven the development of more eco-friendly alternatives (see Fig. 24). The rise of biodegradable coatings has not only been spurred by the need to reduce pollution but also by growing demands for sustainable materials in industries such as packaging, automotive, textiles, and healthcare.^193,194^

One of the primary innovations in biodegradable coatings is the use of natural polymers like chitosan, alginate, and cellulose derivatives, which offer inherent biodegradability without compromising performance. Chitosan, derived from chitin (a biopolymer found in the shells of crustaceans), has shown significant promise as an antimicrobial and biodegradable coating material. Coatings made from chitosan have been successfully applied in food packaging, providing antimicrobial properties that help preserve food without the environmental cost associated with traditional plastic packaging. This polymer is also used in medical applications, such as wound dressings, where it eases healing through its natural antimicrobial action while being completely biodegradable within the body. Research has proven that chitosan coatings can keep over 80% of their antimicrobial efficacy for up to 60 days, making them practical alternatives to synthetic coatings.^195,196^

Another innovative class of biodegradable coatings involves the use of plant-based compounds, particularly those derived from vegetable oils like soybean, castor, or linseed oil. These oils can be chemically changed to form polymers with excellent adhesion properties while also being biodegradable. For example, linseed oil-derived coatings, when polymerized through a process known as oxidative crosslinking, offer a durable, water-resistant finish for use in wood and textile applications. These coatings break down naturally through microbial activity when exposed to the environment, posing no risk of long-term accumulation in ecosystems. Linseed oil coatings, which have been extensively tested for their biodegradability, show complete degradation within three to five months in soil, compared to several years for synthetic alternatives like polyurethane.^197,198^

The integration of bio-based polyesters (see Fig. 24), such as polylactic acid (PLA), polyhydroxyalkanoates (PHA), and poly (butylene succinate) (PBS), into coatings, stands for another breakthrough in biodegradable materials. PLA, derived from renewable resources like corn starch or sugarcane, is one of the most widely studied biodegradable polymers. It is known for its low toxicity and ability to degrade under environmental conditions through hydrolysis. PLA-based coatings are used in a variety of applications, from food packaging to agricultural films, where their biodegradability is crucial to reducing waste. Recent advancements have focused on improving the mechanical properties and thermal stability of PLA-based coatings through the incorporation of plasticizers or reinforcing agents such as nanocellulose or natural fibers. These modifications enhance the performance of the coatings and ensure that they keep their biodegradability and non-toxic characteristics throughout their life.^199,200^

In addition to these plant-based and biopolymer approaches, the use of natural clay minerals, such as montmorillonite or kaolin, has gained attention in the development of biodegradable coatings. These materials are inherently non-toxic and can be combined with biopolymers to improve the mechanical properties of coatings. For instance, when mixed with chitosan or cellulose, clays enhance the structural integrity and water resistance of the coatings while still allowing for natural degradation. Research has shown that the inclusion of 5–10 wt% montmorillonite in a chitosan-based coating improves tensile strength by 50% and significantly reduces water vapor permeability, making it suitable for functional coatings in packaging applications.^201,202^

Recent research also focuses on incorporating enzymatic degradation into biodegradable coatings, where enzymes are integrated into the polymer matrix to accelerate breakdown under specific environmental conditions. This approach allows coatings to degrade in a controlled manner when exposed to moisture or heat, ensuring that they do not persist in landfills or waterways. For example, the use of enzymes like laccase and cellulase in combination with biodegradable polymers such as PHA has been shown to increase the degradation rate of coatings in both soil and compost environments. These coatings offer an eco-friendly alternative to conventional plastic films and are particularly useful in applications where quick degradation is essential, such as agricultural mulching films.^203,204^

Furthermore, a significant focus of current research is on reducing the toxicity of biodegradable coatings by using non-toxic, natural antimicrobial agents instead of metals like silver or copper, which have been associated with eco-toxicity. Natural antimicrobial agents, such as extracts from green tea, thyme, or oregano, are gaining popularity as safe alternatives. These compounds offer antimicrobial protection without leaching harmful substances into the environment. For instance, coatings incorporating green tea extracts have shown up to 95% reduction in bacterial growth on textiles while staying biodegradable and non-toxic in the environment. This shift toward plant-based antimicrobial agents reflects a broader trend in materials science, where the focus is on using non-toxic, renewable resources to develop safer, more sustainable materials.^205,206^

### 7.2 Health and safety concerns

#### 7.2.1 Toxicity in end-use scenarios

Ensuring consumer safety in products that meet the skin is a growing priority in industries such as textiles, cosmetics, and healthcare. In recent years, advancements in material science and toxicology have led to a deeper understanding of how chemicals and compounds in consumer products can affect skin health, particularly when exposure is prolonged or frequent. A primary concern in such products is the potential for chemical leaching from materials like clothing, personal care items, and medical devices. The skin is the largest organ in the human body, and its ability to absorb harmful chemicals or compounds can lead to adverse health effects, including skin irritation, allergies, or even systemic toxicity.^207,208^

In textiles, for example, the use of synthetic dyes, flame retardants, and antimicrobials has raised concerns about their potential to leach into the skin. A key development in ensuring consumer safety has been the shift towards using non-toxic, skin-friendly alternatives to these chemicals. For example, traditional synthetic dyes, which often hold azo compounds linked to skin sensitivity and carcinogenicity, are increasingly being replaced by plant-based dyes derived from natural sources such as indigo, turmeric, or madder root. These natural dyes not only offer reduced toxicity but also come with lower environmental impacts, as they do not need harsh chemicals during their production. Additionally, the use of biopolymers such as polylactic acid (PLA) in fabrics is becoming more common, as PLA is a non-toxic, biodegradable material that does not leach harmful substances into the skin. Research has shown that PLA fabrics, when tested for skin compatibility, show no irritation or adverse reactions in human skin models, even after extended wear.^209,210^

In the realm of personal care products, the move towards “clean” beauty and skincare has led to significant innovations in formulations designed to avoid potentially harmful chemicals like parabens, phthalates, and formaldehyde-releasing agents. Studies have shown that these substances can disrupt endocrine function and cause skin irritation, leading to a growing demand for formulations that are free from such harmful chemicals. The adoption of natural preservatives, like vitamin E (tocopherol), as an alternative to synthetic chemicals has been particularly successful in ensuring that skincare products stay effective without compromising consumer safety. Additionally, there is a growing trend in using plant-based compounds such as aloe vera, chamomile, and calendula in formulations, as these natural ingredients are known for their soothing, anti-inflammatory, and healing properties. These innovations aim not only to protect skin from irritation but also to prevent the accumulation of harmful substances that can lead to long-term health issues.^211,212^

Medical devices that come into direct contact with the skin, such as wound dressings, bandages, and transdermal drug delivery systems, have also become the focus of rigorous safety standards. The materials used in these devices must be biocompatible, ensuring that they do not cause adverse skin reactions, such as allergic dermatitis, or help the leaching of toxic compounds. Recent advancements have led to the development of hydrogels and bio-based polymers that are both safe and effective for use in medical devices. For instance, hydrocolloid-based dressings have gained popularity for their ability to provide a moist wound-healing environment while being gentle on the skin. These materials are made from non-toxic, skin-safe compounds like carboxymethyl cellulose (CMC), which do not release harmful by-products when in contact with skin. Moreover, transdermal patches used for drug delivery are being developed using safe, biocompatible adhesives and polymers like ethyl cellulose, which ensures that the active pharmaceutical ingredients are delivered efficiently without causing irritation or toxic buildup in the skin.^210,213^

One critical area of focus in ensuring consumer safety is the development of “dermatologically tested” products that have undergone comprehensive skin irritation and sensitization testing. These tests, such as the patch test, assess how materials react when applied directly to human skin over extended periods, providing insights into their safety in real-world use. As consumer demand for safer products grows, regulatory bodies have tightened guidelines to require that products not only undergo dermatological testing but also comprehensive toxicity screenings to rule out long-term health risks. These screenings often involve assessing the potential for skin absorption of harmful chemicals or the release of volatile organic compounds (VOCs), which can contribute to skin irritation or allergic reactions.^214,215^

In addition to these advancements, the rise of “green chemistry” has played a crucial role in ensuring that the materials and chemicals used in skin-contact products are safer for both consumers and the environment. Green chemistry focuses on designing chemical products and processes that minimize hazardous substances, and this approach has led to the development of safer alternatives to conventional chemical treatments. For instance, researchers are exploring the use of bio-based surfactants made from renewable plant sources to replace harsh, synthetic surfactants commonly found in soaps, shampoos, and other personal care products. These bio-based surfactants, such as those derived from coconut or corn, are milder on the skin, less likely to cause irritation and degrade more quickly in the environment. They also minimize the risk of skin absorption of toxic compounds, offering a safer option for consumers looking to avoid potentially harmful chemicals.^216,217^

Furthermore, advancements in nanotechnology have opened new possibilities for creating safer, more effective skin contact products. Nanoparticles, when used in cosmetics and personal care products, can enhance the delivery of active ingredients while reducing the need for potentially harmful chemicals.^218^ For instance, nano-encapsulated sunscreens have been developed to provide broad-spectrum UV protection without the risk of skin irritation or toxicity associated with traditional chemical sunscreens. These nano-formulations, made from zinc oxide or titanium dioxide, are safe for skin contact and remain on the surface of the skin, preventing deeper absorption and potential toxicity.^219,220^

#### 7.2.2 Compliance with safety standards

In recent years, regulatory aspects surrounding product safety, particularly for materials that meet consumers, have become increasingly stringent across international markets. This shift has been primarily driven by heightened awareness of the potential risks associated with chemicals and compounds used in consumer products, including their toxicity, environmental impact, and long-term health effects. As a result, regulatory bodies around the world have introduced more rigorous safety standards, compelling manufacturers to adapt to evolving frameworks aimed at ensuring consumer protection while also promoting sustainability.^221,222^

In the European Union, Regulation (EC) no 1907/2006, known as REACH (Registration, Evaluation, Authorisation, and Restriction of Chemicals), plays a central role in regulating the safety of chemicals used in consumer products, including coatings, textiles, and cosmetics. REACH requires manufacturers to provide detailed safety data on the chemicals they use, ensuring that only substances deemed safe are allowed on the market. For example, under REACH, substances like bisphenol A (BPA), which has been linked to endocrine disruption, are subject to rigorous restrictions due to concerns over their long-term health effects. Similarly, REACH also restricts the use of certain flame retardants and dyes that are known to cause skin irritation or carcinogenicity. These regulations have led to the development of safer alternatives, such as flame retardants derived from phosphorus or boron compounds, which provide comparable performance without the associated health risks.^223,224^

The United States has its own set of regulations governed by the Environmental Protection Agency (EPA) and the Food and Drug Administration (FDA), which oversee the safety of chemicals in consumer products. The Toxic Substances Control Act (TSCA) of 1976 was significantly amended in 2016, enhancing the EPA's authority to evaluate and regulate chemicals in commercial products. The TSCA requires manufacturers to give safety information on chemicals used in coatings, textiles, and personal care items before they are introduced to the market. As part of this process, products undergo extensive toxicity testing to assess risks such as carcinogenicity, reproductive toxicity, and skin sensitization. Recent amendments to the TSCA have focused on high-priority chemicals, including phthalates and formaldehyde-based resins, both of which have been found to have adverse health effects with prolonged exposure. The introduction of non-toxic, biobased alternatives, such as polyols derived from renewable resources, has been a direct result of these regulatory pressures.^225,226^

In Asia, markets like Japan and China have increasingly adopted stricter regulations to ensure consumer safety. Japan's Chemical Substances Control Law (CSCL) requires the registration of all chemical substances used in consumer products, and manufacturers must ensure that their products follow safety standards set by the Japan Ministry of Health, Labour and Welfare (MHLW). The use of toxic substances such as lead-based compounds in textiles and coatings is strictly checked, and products holding chemicals like toluene, a solvent with known health risks, are banned unless safe alternatives are used. China, through its National Standardization Administration, has developed a set of guidelines known as the GB (Guobiao) standards, which address the safety of chemicals in products like textiles, food packaging, and cosmetics. Under these regulations, manufacturers must follow restrictions on heavy metals, harmful dyes, and volatile organic compounds (VOCs), ensuring that consumer products are free from toxic substances that may pose health risks.^227,228^

A particularly important aspect of these regulations is their focus on consumer exposure during the end-use phase. For instance, in textiles, ensuring that clothing or upholstery does not release harmful chemicals into the environment or come into direct contact with the skin is a key concern. Standards such as the Global Organic Textile Standard (GOTS) have set global benchmarks for textile production that require manufacturers to use only non-toxic dyes, flame retardants, and finishes, ensuring that these materials do not degrade into hazardous by-products. GOTS-certified textiles have been found to be free from harmful chemicals such as azo dyes and formaldehyde, which are known to cause allergic reactions, skin irritation, and long-term health problems. Similarly, the OEKO-TEX Standard 100 certification ensures that textiles meet stringent criteria for chemical safety, with products undergoing tests for a range of harmful substances, including heavy metals, pesticides, and phthalates. These certifications have made it easier for consumers to name products that meet high safety standards, promoting trust and confidence in the marketplace.^229,230^

Furthermore, regulatory standards are also addressing the environmental impacts of product life cycles, pushing manufacturers towards sustainable practices.^231^ In the European Union, the EcoDesign Directive, which focuses on making products more environmentally friendly throughout their life cycles, requires manufacturers to consider the end-of-life disposal of products. This includes ensuring that products are either recyclable or biodegradable and that harmful substances are minimized or eliminated from the product. In the context of coatings and textiles, this means that manufacturers are encouraged to develop materials that can degrade without releasing toxic chemicals into the environment. For example, bio-based coatings and textiles, which use plant-derived materials that are safe for both human health and the environment, are becoming more common as regulatory pressures increase.^192,232^

The regulatory landscape continues to evolve as new chemicals are discovered and emerging health concerns arise. Manufacturers are facing increasing pressure to ensure that their products follow these safety standards, and the consequences of non-compliance can be significant. For instance, a company found to be using banned chemicals such as perfluorooctanoic acid (PFOA) in textile coatings may face large fines, recalls, and reputational damage. This has led to a growing emphasis on compliance with safety standards, as well as proactive research and development of safer, non-toxic alternatives.^233,234^

### 7.3 Regulatory and compliance standards

#### 7.3.1 Existing regulations

In the ever-evolving landscape of product safety and environmental protection, safety standards like OEKO-TEX and REACH have played pivotal roles in shaping the regulatory environment for chemicals used in textiles, coatings, and consumer products. These standards aim to mitigate potential risks to human health and the environment by establishing comprehensive guidelines for chemicals in manufacturing processes. They not only address concerns over toxicity and ecological impact but also respond to consumer demands for transparency and the reduction of harmful substances in products. As a result, these regulations have fostered innovation in the development of safer materials and production techniques that align with growing sustainability goals.^235,236^

The OEKO-TEX Standard 100 is a globally recognized certification system for textiles and fabrics, which ensures that products are free from harmful levels of toxic substances. The standard focuses on several key areas, such as chemical safety, ecological impact, and human health, by requiring manufacturers to follow stringent testing criteria. This includes testing for a wide range of chemicals, such as azo dyes, formaldehyde, phthalates, pesticides, and heavy metals like lead, cadmium, and mercury. For example, OEKO-TEX certification ensures that textiles do not hold more than 0.1% of certain harmful azo dyes, which are linked to allergic reactions and skin irritation. Additionally, the limit for formaldehyde in textiles is capped at 75 parts per million (ppm), reducing the risk of skin irritation and respiratory issues that can result from prolonged exposure to this carcinogenic compound. By setting up such thresholds, OEKO-TEX has empowered consumers to make informed choices about the products they buy, knowing that they meet high safety standards.^237,238^

In Europe, the REACH (Registration, Evaluation, Authorization, and Restriction of Chemicals) regulation is a cornerstone of chemical safety in consumer products, including coatings, textiles, and cosmetics. REACH aims to improve the protection of human health and the environment from the risks posed by chemicals while also enhancing the competitiveness of the European chemicals industry. Under REACH, companies must register the chemicals they use in products, provide safety data, and show that the substances they manufacture, or import meet stringent safety requirements. A key feature of REACH is its focus on substances of very high concern (SVHCs), such as endocrine disruptors, carcinogens, and persistent chemicals that accumulate in ecosystems. For example, the regulation has led to restricting substances like cadmium, which is commonly used in pigments and can accumulate in the body over time, posing long-term health risks. The inclusion of REACH-compliant alternatives like safer, non-toxic pigment compounds has led to the formulation of coatings and textiles that offer the same performance without compromising safety.^239,240^

Under REACH, manufacturers also must assess the risks associated with chemicals based on their potential for human exposure, ensuring that only chemicals that do not pose a significant risk to human health are used in consumer products. For example, in the textile industry, chemicals used for finishing treatments, like flame retardants or water repellents, are closely regulated. Certain flame retardants, such as polybrominated diphenyl ethers (PBDEs), have been shown to have neurotoxic effects and are restricted under REACH. As a result, manufacturers are turning to alternative, safer flame retardants, such as phosphorus-based compounds or organoboron compounds, which offer similar fire-resistance properties but with less environmental and health impact. The adoption of these safer alternatives proves how REACH's regulatory framework has driven innovation in safer materials and chemicals.^241,242^

One of the most significant advancements in chemical safety and regulation has been the introduction of transparency requirements within both OEKO-TEX and REACH. The global demand for greater product transparency has resulted in consumers expecting more information about the chemicals present in the products they use. Both standards require manufacturers to show any chemicals used in textiles and coatings production, allowing consumers to make informed decisions about the products they purchase. This transparency has been especially important in the cosmetics and personal care industry, where consumers are increasingly concerned about the presence of ingredients such as parabens, phthalates, and sulfates, which have been linked to endocrine disruption and skin irritation. The push for more transparency has not only led to safer products but also fostered a consumer-driven market that prioritizes eco-friendly, non-toxic materials.^243,244^

Beyond these safety standards, the push for regulatory frameworks has also extended to the environmental impact of chemicals used in products. Regulations like REACH focus on ensuring that the chemicals used in consumer products do not contribute to long-term environmental damage. This includes ensuring that chemicals used in coatings and textiles break down naturally in the environment, reducing the potential for soil and water contamination. For instance, in the case of water-repellent treatments, manufacturers have moved towards using perfluorocyclopropane (PFCA)-free alternatives, which do not persist in the environment like their perfluorinated counterparts. The shift toward more biodegradable and environmentally friendly substances has been an essential part of aligning with evolving global sustainability goals.^245,246^

Another important consideration in existing regulations is the continuous monitoring and updating of safety standards based on emerging scientific data. As research advances, new compounds and materials are regularly evaluated for their safety profiles, and regulatory frameworks are updated accordingly. For example, the identification of new endocrine-disrupting chemicals (EDCs) has led to stricter control over their use in consumer products. The recognition of bisphenol S (BPS) as an EDC, for instance, has prompted several manufacturers to phase it out in favor of safer alternatives, such as bio-based plasticizers derived from renewable resources like castor oil. The ongoing review and updating of safety standards ensure that the regulations are still relevant in addressing the latest scientific findings, keeping consumer safety at the forefront.^247,248^

#### 7.3.2 Challenges with new coatings

As industries continue to innovate in materials science, the development of new coatings and functional materials has introduced a range of complex compliance challenges. These challenges are largely driven by the evolving nature of regulations that aim to balance technological advancement with the protection of human health and the environment. The emergence of novel materials, particularly in fields like textiles, construction, and automotive industries, often requires manufacturers to navigate a complex regulatory landscape, ensuring that their products meet the required safety, durability, and environmental standards. These compliance issues can become particularly complicated when new materials do not fall neatly into established regulatory categories, necessitating new testing methods and risk assessments to ensure their safety.^249,250^

One of the key challenges faced by manufacturers of innovative coatings is ensuring that these new materials comply with stringent regulatory requirements, such as those outlined in REACH (Registration, Evaluation, Authorization, and Restriction of Chemicals) and the Toxic Substances Control Act (TSCA). For example, newer flame-retardant coatings that rely on phosphorus-based compounds or organoboron compounds may not have been extensively tested under existing regulations. This can create uncertainty about their safety profiles, as these materials may not have been fully assessed for their long-term effects on human health and the environment. In some cases, the regulatory bodies may require extensive data on the chemical structure, degradation products, and exposure pathways of these materials, which can delay product launch and increase research and development costs. The approval process for such new substances often involves a comprehensive review of their potential toxicity, environmental impact, and biodegradability. Manufacturers must conduct additional research, often including animal testing, lifecycle assessments, and environmental impact evaluations, to ensure that their products comply with regulatory standards.^251,252^

Another complication arises when new materials and coatings contain a combination of functional properties, such as antimicrobial, flame-retardant, and water-repellent features. These multi-functional coatings pose unique challenges in terms of ensuring compatibility with regulatory guidelines. For instance, certain antimicrobial agents, such as silver nanoparticles, have been found to exhibit toxicological effects, including cytotoxicity and genotoxicity, when present in high concentrations.^253,254^ As regulations around the use of nanomaterials become more stringent, manufacturers must ensure that their multi-functional coatings comply with new rules that govern the safety of nano-sized particles. In the European Union, the European Chemicals Agency (ECHA) has implemented specific guidelines for the safety of nanomaterials under REACH, requiring manufacturers to provide detailed data on the physicochemical properties of nanoparticles, their release potential, and their long-term impacts on both human health and the environment. Similarly, in the United States, the Environmental Protection Agency (EPA) has been active in reviewing the safety of nanomaterials used in consumer products, leading to the imposition of stricter guidelines on the production and use of such materials. The need to evaluate new coatings for their potential release of nanoparticles adds an additional layer of complexity to the compliance process, often requiring specialized testing and risk assessments.^255,256^

In addition to the concerns surrounding health and safety, regulatory compliance for innovative coatings also extends to environmental sustainability. As the global focus on sustainability intensifies, manufacturers are increasingly expected to develop products that not only meet safety standards but also minimize their environmental footprint. This is especially true for coatings used in textiles and construction materials, where concerns about the release of hazardous substances into the environment during both production and end-of-life disposal have come to the forefront. For example, certain flame retardants and water-repellent chemicals, such as perfluorooctane sulfonate (PFOS) and perfluorooctanoic acid (PFOA), are persistent in the environment and can accumulate in living organisms, leading to long-term ecological harm.^257^ The restriction of these substances under regulations such as the Stockholm Convention on Persistent Organic Pollutants (POPs) has forced manufacturers to seek alternative flame-retardant or water-repellent chemicals that are less harmful to the environment. However, finding such alternatives that are both effective and compliant with regulatory standards can be a significant challenge. For instance, while phosphorus-based flame retardants have gained popularity as safer alternatives, their efficacy and environmental impact must still be thoroughly assessed, which often requires additional testing and data collection.^258,259^

The evolving nature of global regulations also creates difficulties for innovative coatings manufacturers in international compliance. As safety standards and environmental regulations vary widely between regions, manufacturers must navigate a patchwork of requirements to ensure their products are compliant in multiple markets. In some cases, a coating that is approved for use in one region may not meet the safety or environmental standards required in another. For example, while certain antimicrobial agents like triclosan have been banned in Europe due to their environmental toxicity, they may still be permitted in certain applications in other regions like North America, albeit under specific guidelines. This disparity in regulatory frameworks can create significant challenges for manufacturers who are looking to market their products globally, as they must not only ensure their products meet the requirements of each individual market but also consider the costs and logistical hurdles associated with compliance in multiple regions. This complexity is further exacerbated by the fast-paced pace of regulatory change, as new chemicals and materials are continuously being evaluated and restricted based on emerging research.^248,260^

Lastly, as the demand for sustainable and biodegradable coatings increases, there are additional challenges regarding the development of coatings that meet these criteria while still offering the required performance characteristics. Biodegradable coatings, particularly those used in food packaging and textiles, face stringent requirements regarding their ability to break down safely without leaving behind harmful residues.^261,262^ Innovations in this area, such as the use of plant-based polymers or biopolymers like polylactic acid (PLA), offer promising alternatives to traditional synthetic coatings. However, these new materials must undergo rigorous testing to ensure that they meet the durability, performance, and safety standards required for end-use applications. Furthermore, there are often challenges in proving that biodegradable coatings do not contribute to microplastic pollution, a growing concern in environmental science. The research into developing non-toxic, eco-friendly alternatives to traditional synthetic coatings has been promising, but the regulatory landscape for these materials is still evolving, requiring manufacturers to stay ahead of the curve in terms of compliance.^263–265^

## 8. Future directions and research needs

Although environmentally friendly textile coating substitutes are making great strides, they frequently fall short of conventional chemical coatings in terms of usefulness, scalability, and durability. This discrepancy emphasizes the necessity of ongoing research and development to improve sustainable coatings' properties for wider industrial applications.^266,267^ Sustainability must be given top priority when examining potential future directions and research requirements for cutting-edge functional coatings in protective fabrics. The creation of biodegradable flame retardants, for example, can drastically lessen environmental effects while preserving performance criteria. Innovations in sustainable coatings, especially bio-based and renewable alternatives, are essential.^268^ Furthermore, resolving commercialization issues is necessary to guarantee that novel materials may be introduced to the market at a reasonable cost. For these cutting-edge coatings to be widely used, cost-effectiveness and production process scaling will be crucial. Cross-disciplinary research is also essential for promoting innovation in this area.^269^ Working together with textile scientists, material engineers, environmental experts, and regulatory specialists can produce more complete solutions that improve textile functions and support sustainability objectives. Future research can responsibly expand the capabilities of protective textiles by focusing on these areas: interdisciplinary collaboration, commercialization methods, and sustainable innovations.^270,271^Table 3 offers a well-organized summary of the research requirements and future directions for cutting-edge functional coatings in protective fabrics.

**Table 3:** Summary of the research requirements and future directions for cutting-edge functional coatings in protective fabrics

Sections	Details	References
Future directions and research needs	Investigating potential future paths that can improve the sustainability and functionality of textile coatings is essential as the market for high-performance protective textiles keeps changing	Ojstršek *et al.*^25^
		Ielo *et al.*^13^
Innovations in sustainable coatings	Sustainable textile coating solutions are becoming more and more popular as environmental degradation concerns grow. Researchers are working hard to find solutions that satisfy sustainability standards and performance requirements	Aldalbahi *et al.*^272^
Bio-based and renewable coatings	(i) Biodegradable flame retardants: materials that are biodegradable when disposed of such as phosphorylated chitosan made from biomass, have flame-retardant qualities	Smet *et al.*^268^
	(ii) Natural antimicrobials: plant extracts or essential oils have antibacterial qualities without being harmful	
	(iii) Self-cleaning agents: utilizing modified starches or natural waxes can provide self-cleaning qualities, guaranteeing environmental safety as they degrade	
	Research should concentrate on improving these materials for improved performance traits like those of synthetic counterparts, such as resilience and effectiveness in a range of circumstances (*e.g.*, repeated washing)	
Commercialization challenges	Innovative functional coatings have a lot of potential, but before they are widely used in industrial settings, a few obstacles need to be removed	Holme^269^
Scaling and cost efficiency	(i) Creating novel materials frequently requires expensive research that might not be profitable to scale up	Frederichi *et al.*^28^
		Ojstršek *et al.*^25^
	(ii) New configurations or modifications to current industrial infrastructure may be necessary for innovative coating solutions; this shift may necessitate a large financial outlay	Ghazal *et al.*^26^
		Joshi^273^
	(iii) Persuading producers in well-established industries necessitates proving cost-effectiveness and higher performance benefits, which calls for extensive testing against industry standards. To encourage businesses to quickly embrace novel coating technologies, research must focus on creating scalable techniques that strike a balance between creativity and economic viability	
Cross-disciplinary research needs	The intricacy of developing multifunctional coatings demands cooperation between several scientific fields, including engineering, chemistry, material science, and biology, to efficiently utilize a variety of specialized knowledge	Salonitis *et al.*^271^
		Baer *et al.*^274^
Role of collaborative research	(i) Encouraging knowledge exchange: by integrating knowledge from many domains, researchers can create comprehensive solutions that solve several issues, including eco-friendliness and performance improvement	Bhattacharjee *et al.*^270^
	(ii) Developing advanced testing protocols: working together, teams may develop standardized testing procedures tailored to the needs of multifunctional textiles, which is a crucial first step in obtaining the regulatory clearances required for market launch	
	(iii) Promoting innovation hubs: by creating interdisciplinary innovation centers, researchers from academia and industry partners collaborate to solve real-world issues through projects that highlight the application of functional textiles in fields like healthcare logistics supply chains, where emergency preparedness depends on having clothing that is both safe and effective	

## 9. Conclusions

The integration of advanced functional coatings into protective textiles has revolutionized their application across healthcare, industrial, and military sectors, enhancing safety, durability, and hygiene. This review has highlighted the importance of flame retardant, antimicrobial, and self-cleaning coatings, each contributing unique benefits to protective fabrics. Flame-retardant coatings, particularly phosphorus-based and bio-inspired solutions, have proven effective in enhancing fire resistance while addressing environmental concerns associated with halogenated retardants. Antimicrobial coatings, utilizing metal nanoparticles, chitosan, and natural extracts, have provided significant resistance against microbial growth, making them essential for medical and high-risk environments. Self-cleaning coatings based on photocatalytic and superhydrophobic technologies have demonstrated remarkable efficiency in reducing contamination and maintenance efforts, ensuring long-term usability. However, achieving multifunctionality while maintaining mechanical integrity, flexibility, and sustainability remains a significant challenge, requiring innovative approaches in material selection and coating methodologies. The future of protective textiles lies in the development of hybrid coatings that balance multiple functionalities without compromising fabric properties. Research efforts should focus on enhancing coating durability, reducing environmental impact, and improving large-scale manufacturing processes. The incorporation of bio-based, non-toxic materials will be crucial in meeting growing regulatory and sustainability demands. Furthermore, advancements in nanotechnology and smart coatings will enable responsive textiles capable of adapting to environmental changes. Collaboration among scientists, engineers, and industry stakeholders is essential for translating laboratory innovations into commercially viable products. As functional coatings evolve, protective textiles will continue to redefine safety standards, providing enhanced protection in extreme conditions while promoting sustainability and efficiency in material use.

## Data availability

No primary research results, software or code have been included and no new data were generated or analysed as part of this review.

## Author contributions

Conceptualization: J. G.; methodology: J. G., N. S. R., T. R. A. and T. N.; formal analysis: J. G., N. S. R., T. R. A, T. N., V. R. and T. I.; investigation: J. G., N. S. R., T. R. A., T. N.; validation: J. G., N. S. R., T. R. A., T. N., V. R. and T. I.; resources: J. G., N. S. R., T. R. A., T. N.; visualization: J. G., N. S. R., T. R. A, T. N., V. R. and T. I.; supervision: J. G., N. S. R.; V. R. and T. I.; original draft: J. G., N. S. R., T. R. A., T. N., V. R. and T. I.; writing, review, and editing: J. G., N. S. R., T. R. A, T. N., and T. I. All authors reviewed, and approved the final version of the manuscript.

## Conflicts of interest

The authors declared that they don't have any conflict of interest.
