# Correction: Functional coatings for textiles: advancements in flame resistance, antimicrobial defense, and self-cleaning performance

**DOI:** 10.1039/d5ra90049b

**Published:** 2025-05-02

**Authors:** Joyjit Ghosh, Nishat Sarmin Rupanty, Tasneem Noor, Tanvir Rahman Asif, Tarikul Islam, Vladimir Reukov

**Affiliations:** a Department of Textiles, Merchandising, and Interiors, University of Georgia Athens Georgia 30602 USA tarikul@uga.edu; b Department of Textile Engineering, Ahsanullah University of Science and Technology Dhaka 1208 Bangladesh; c Department of Textile Engineering, Jashore University of Science and Technology Jashore 7408 Bangladesh

## Abstract

Correction for ‘Functional coatings for textiles: advancements in flame resistance, antimicrobial defense, and self-cleaning performance’ by Joyjit Ghosh *et al.*, *RSC Adv.*, 2025, **15**, 10984–11022, https://doi.org/10.1039/D5RA01429H.

The authors regret that one of the authors (Vladimir Reukov) was incorrectly marked as a corresponding author in the original article. The sole corresponding author is Tarikul Islam. The corrected author list is as shown here.

The Royal Society of Chemistry apologises for these errors and any consequent inconvenience to authors and readers.

